# Supplementary material for: Nuclear CK1δ as a critical determinant of PER:CRY complex dynamics and circadian period
Source: eLife. 2026 Jun 15;15:RP110786. doi: 10.7554/eLife.110786 (PMC13268647; doi:10.7554/eLife.110786)
Supplement: Figure 1—source data 1. [file elife-110786-fig1-data1.docx]

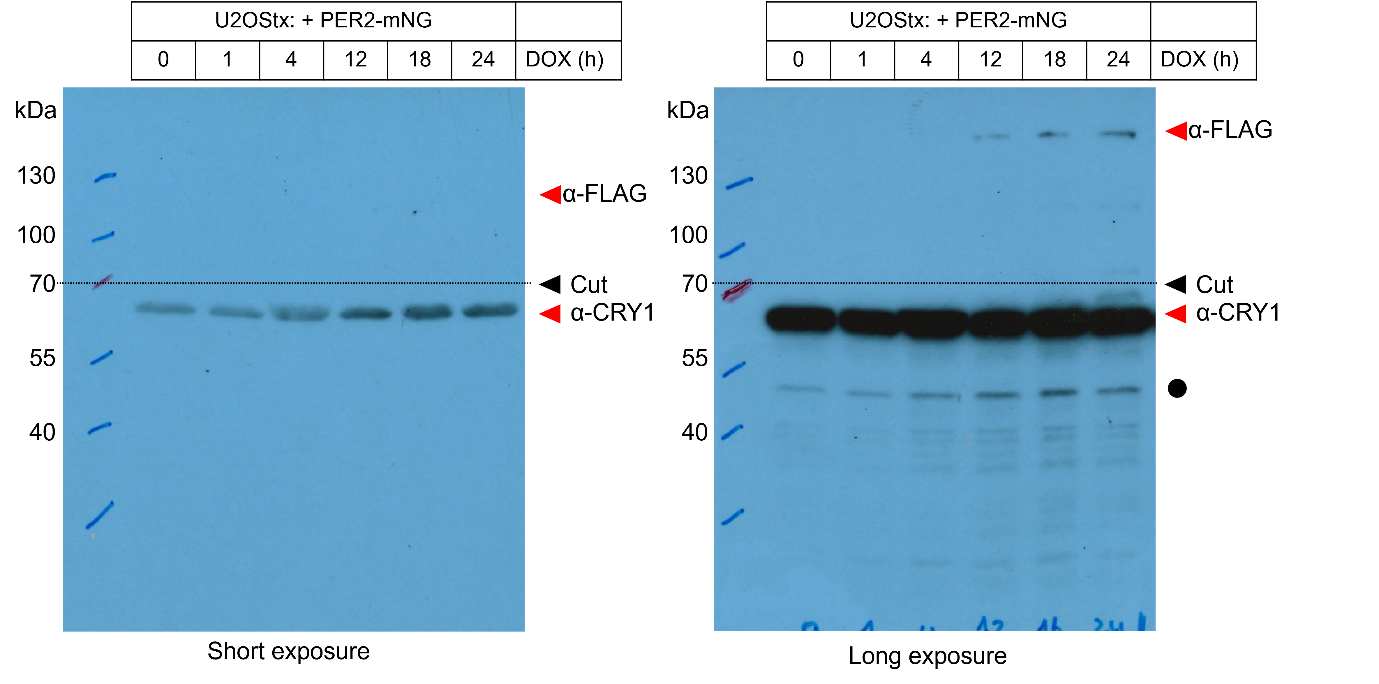


**Figure 1B – Source Data 1.** Original film corresponding to Figure 1B. U2OStx cells were transfected with PER2-mNG, induced with DOX, and protein samples were taken 0, 1, 4, 12, 18, and 24 h post-induction for immunoblotting. The top half of the blot was decorated with anti-FLAG antibody to detect overexpressed PER2-mNG and the lower half was decorated with anti-CRY1 (in-house) to detect endogenous CRY1. The black circle indicates non-specific signal. Both short and long exposures were taken from the same membrane.


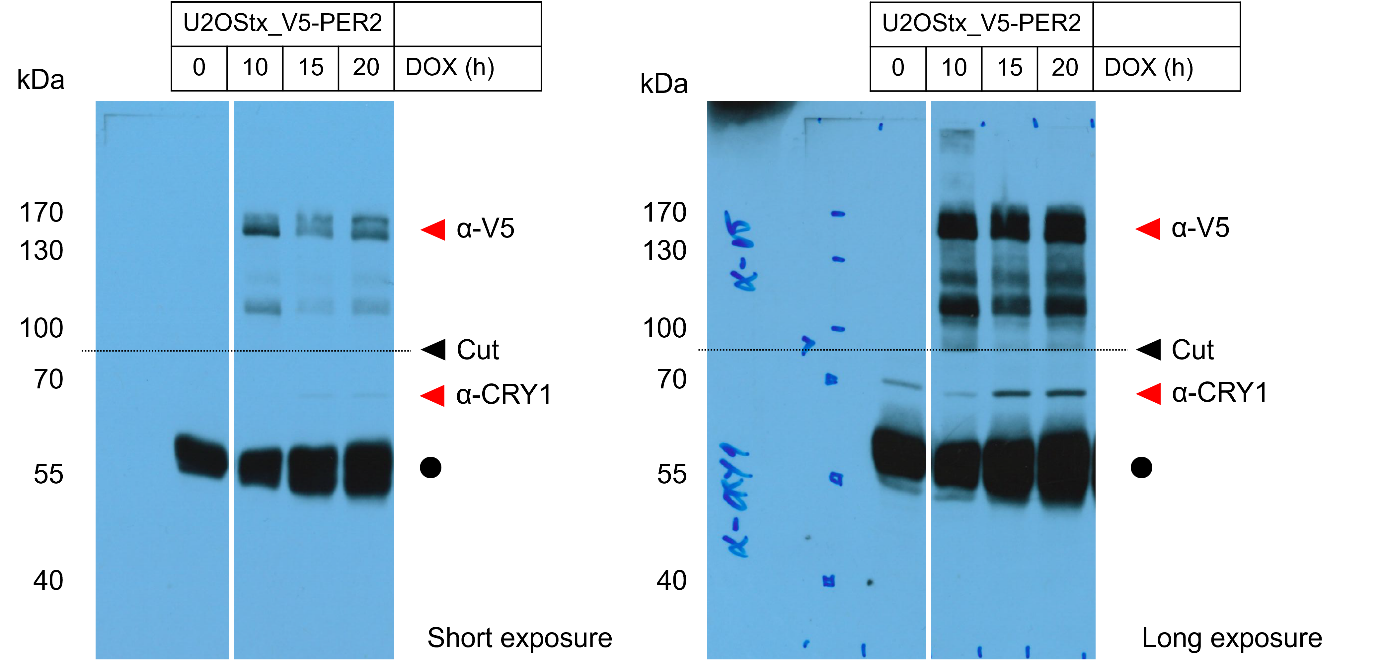


**Figure 1C – Source Data 1.** Original film corresponding to Figure 1C. U2OStx_V5-PER2 cells were induced with DOX and protein samples were taken 0, 10, 15, and 20 h post-induction for immunoblotting. The top half of the blot was decorated with anti-V5 antibody to detect overexpressed V5-PER2 and the lower half was decorated with anti-CRY1 (in-house) to detect endogenous CRY1. The black circle indicates non-specific signal. Both short and long exposures were taken from the same membrane.
