# Supplementary figures and images for: Nuclear CK1δ as a critical determinant of PER:CRY complex dynamics and circadian period
Source: eLife. 2026 Jun 15;15:RP110786. doi: 10.7554/eLife.110786 (PMC13268647; doi:10.7554/eLife.110786)

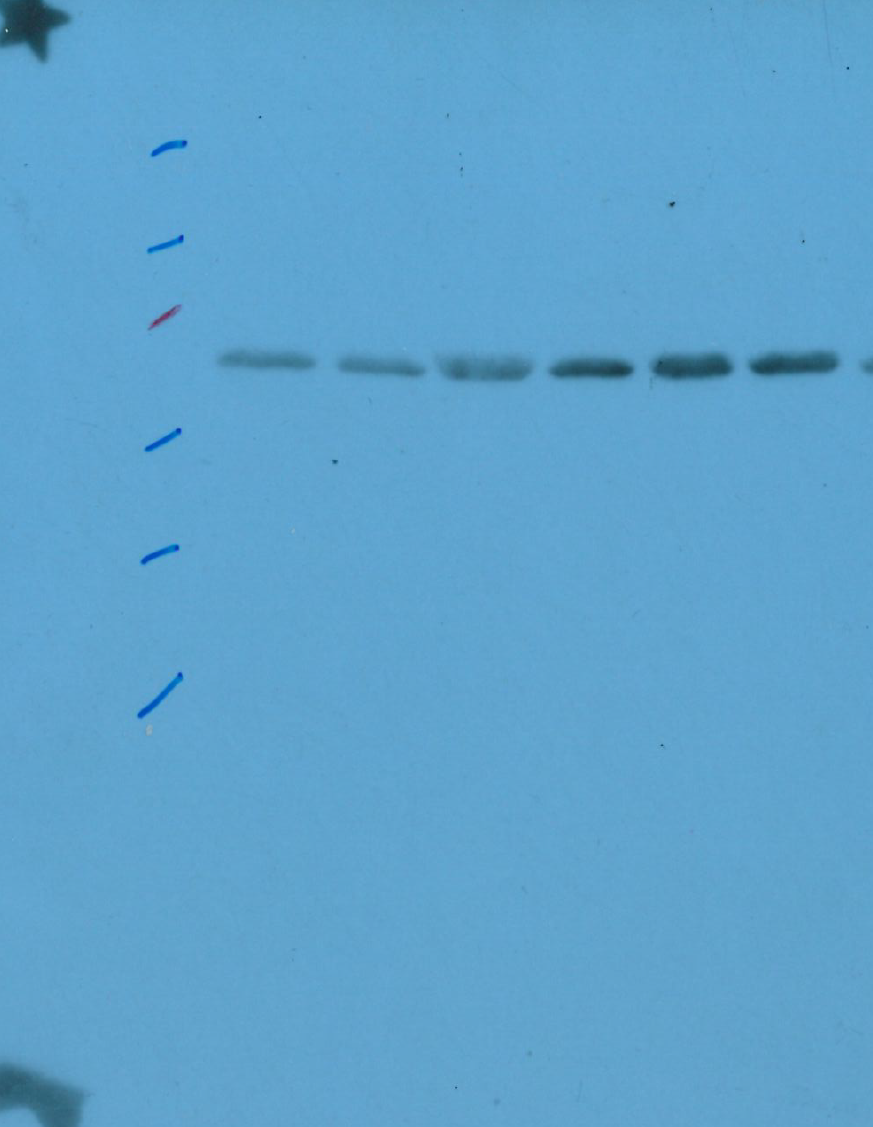

Supplement: Figure 1—source data 2. [file elife-110786-fig1-data2.zip › Fig 1_SD/Figure 1B_endoCRY1.tif]

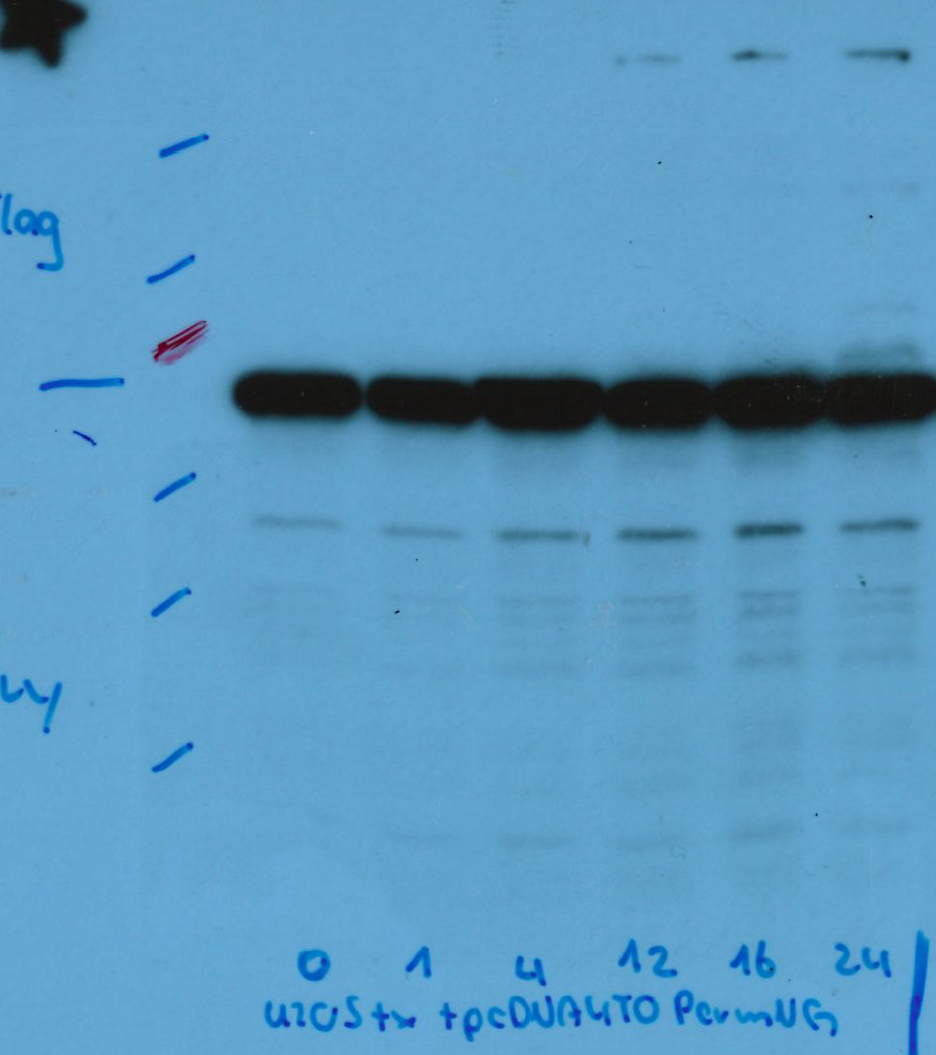

Supplement: Figure 1—source data 2. [file elife-110786-fig1-data2.zip › Fig 1_SD/Figure 1B_FLAG.tif]

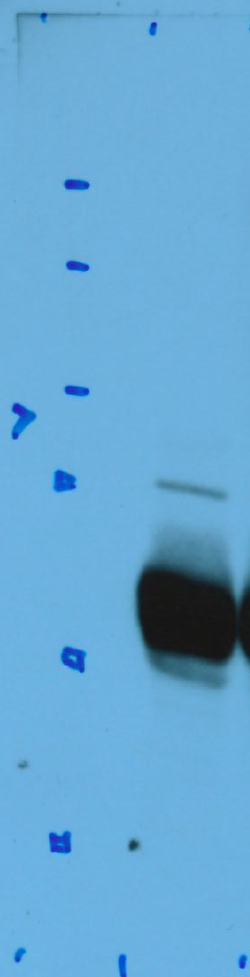

Supplement: Figure 1—source data 2. [file elife-110786-fig1-data2.zip › Fig 1_SD/Figure 1C_long1.tif]

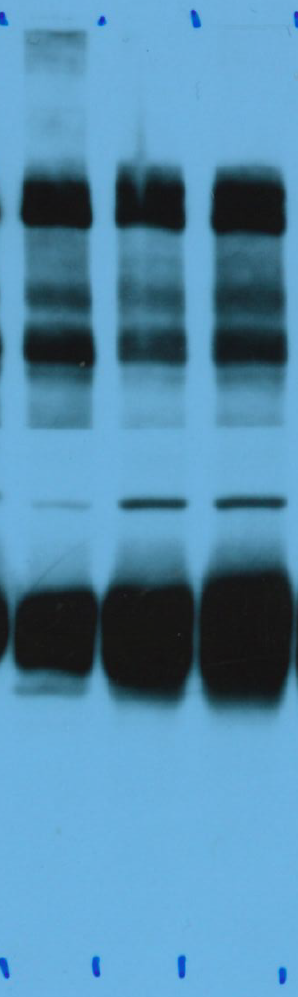

Supplement: Figure 1—source data 2. [file elife-110786-fig1-data2.zip › Fig 1_SD/Figure 1C_long2.tif]

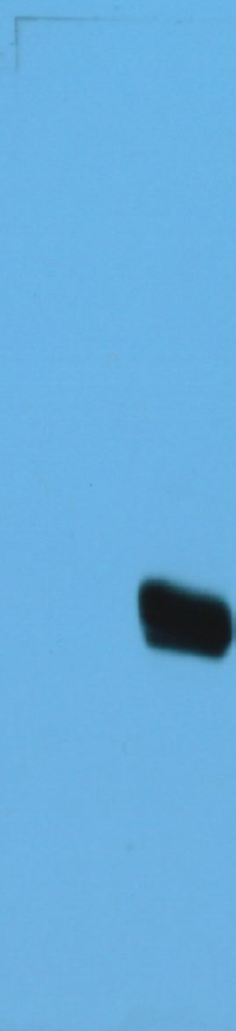

Supplement: Figure 1—source data 2. [file elife-110786-fig1-data2.zip › Fig 1_SD/Figure 1C_short1.tif]

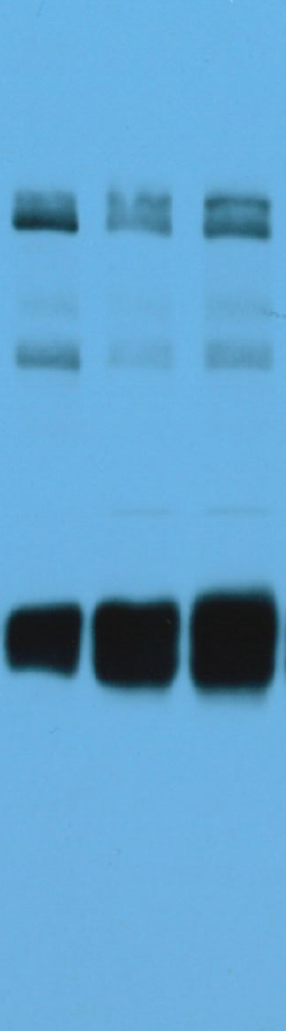

Supplement: Figure 1—source data 2. [file elife-110786-fig1-data2.zip › Fig 1_SD/Figure 1C_short2.tif]

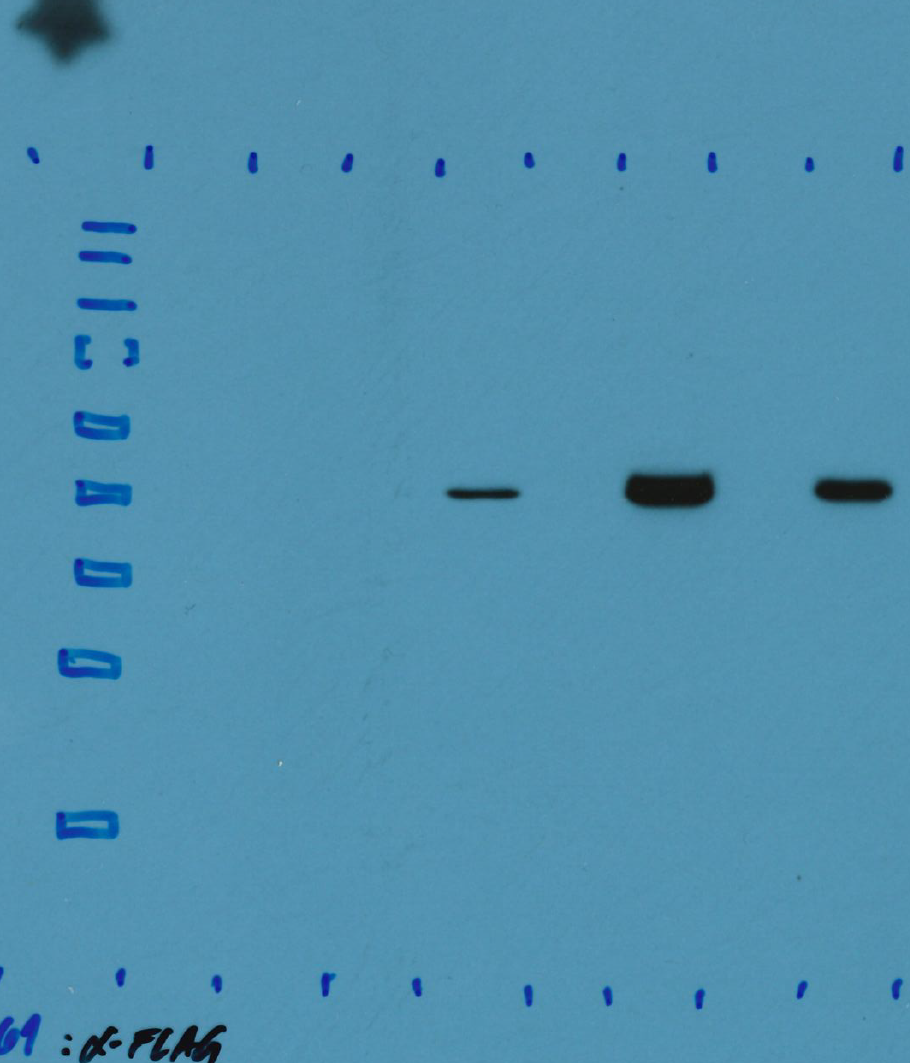

Supplement: Figure 3—source data 2. [file elife-110786-fig3-data2.zip › Fig 3_SD/Figure 3B_1_FLAG.tif]

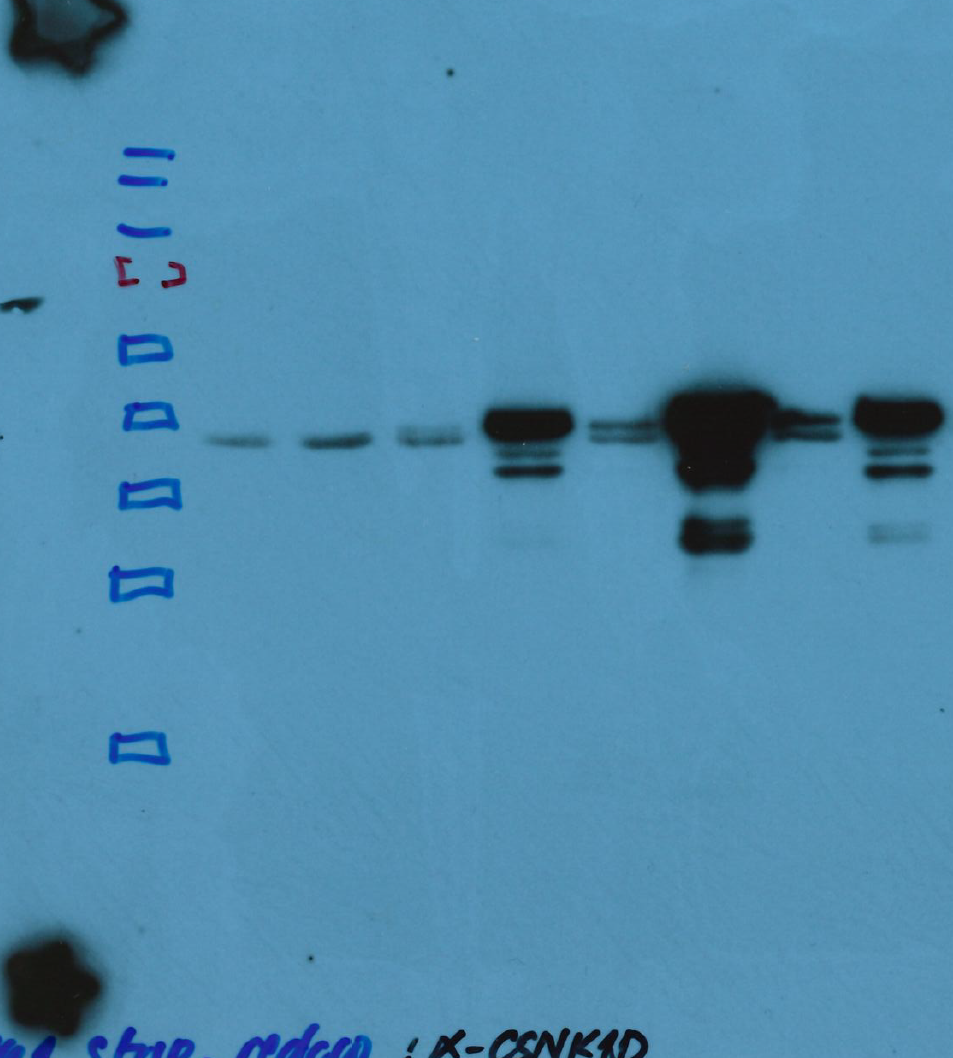

Supplement: Figure 3—source data 2. [file elife-110786-fig3-data2.zip › Fig 3_SD/Figure 3B_2_CK1d.tif]

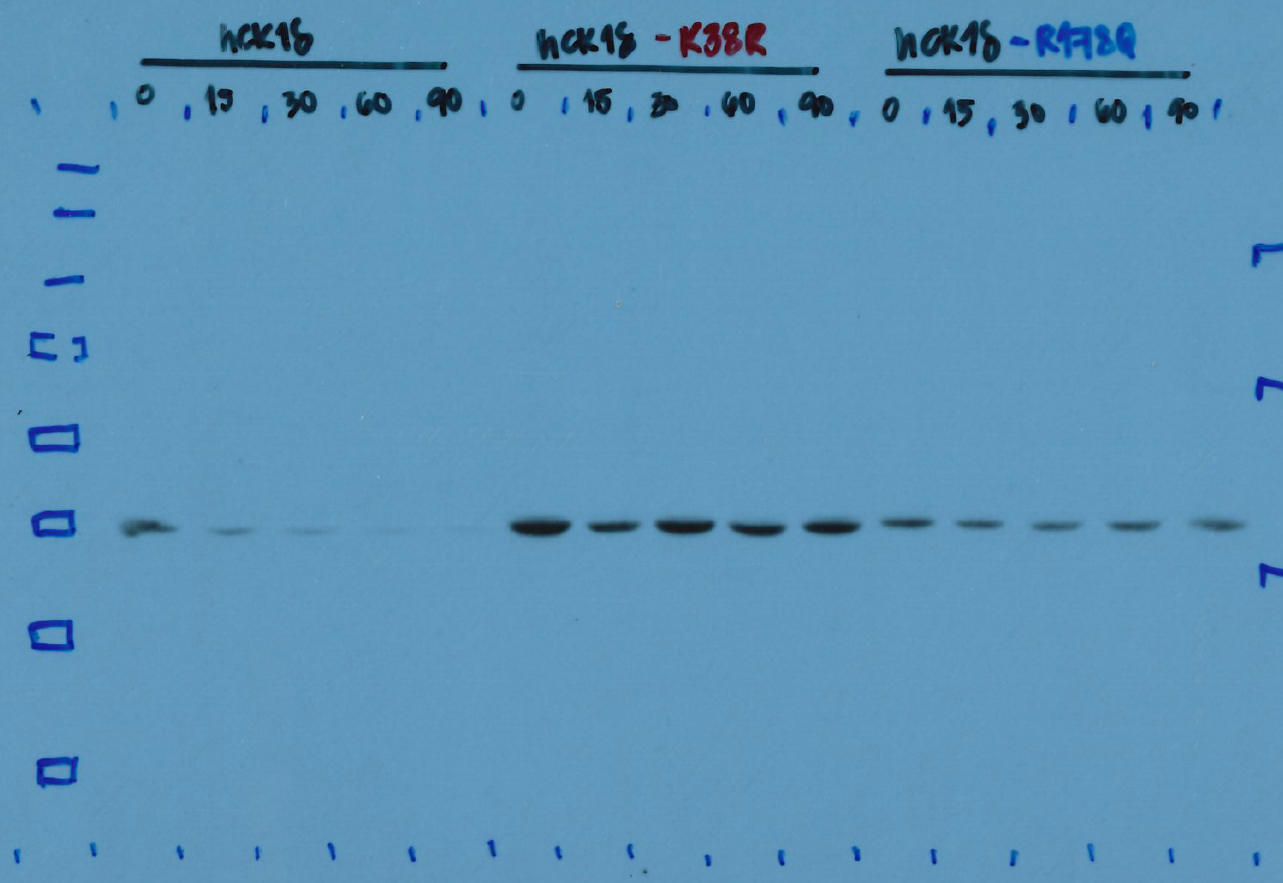

Supplement: Figure 3—source data 2. [file elife-110786-fig3-data2.zip › Fig 3_SD/Figure 3C_CHX_FLAG.tif]

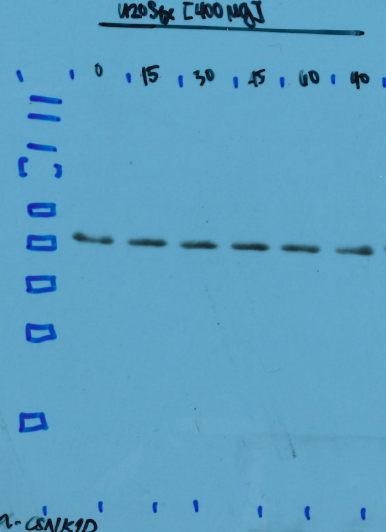

Supplement: Figure 3—source data 2. [file elife-110786-fig3-data2.zip › Fig 3_SD/Figure 3D_CHX_endoCK1.tif]

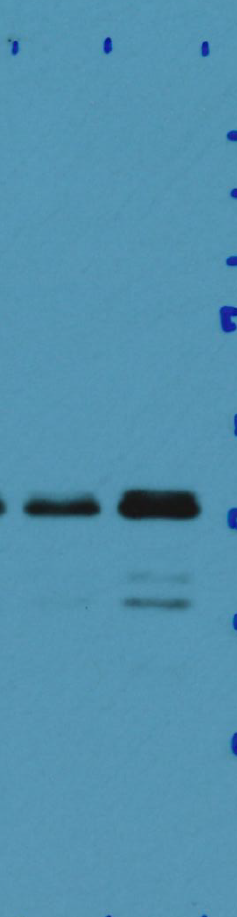

Supplement: Figure 3—source data 2. [file elife-110786-fig3-data2.zip › Fig 3_SD/Figure 3F_1_oxCK1.tif]

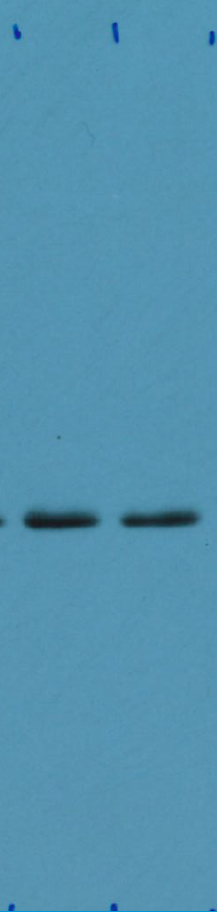

Supplement: Figure 3—source data 2. [file elife-110786-fig3-data2.zip › Fig 3_SD/Figure 3F_2_endoCK1.tif]

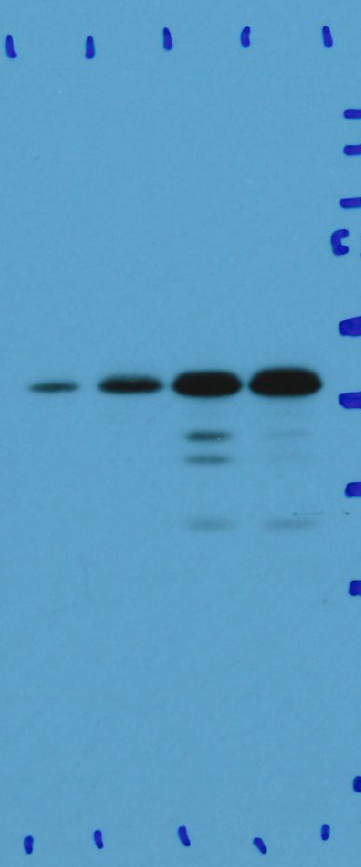

Supplement: Figure 3—source data 2. [file elife-110786-fig3-data2.zip › Fig 3_SD/Figure 3G_1_oxCK1.tif]

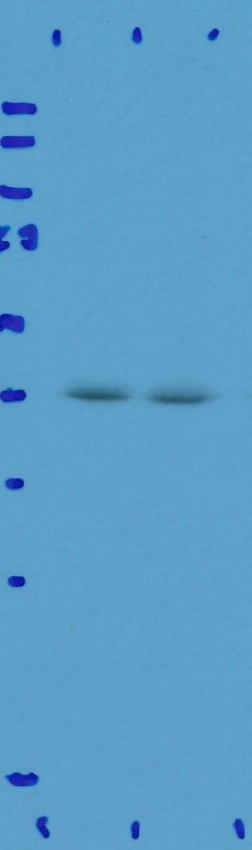

Supplement: Figure 3—source data 2. [file elife-110786-fig3-data2.zip › Fig 3_SD/Figure 3G_2_endoCK1.tif]

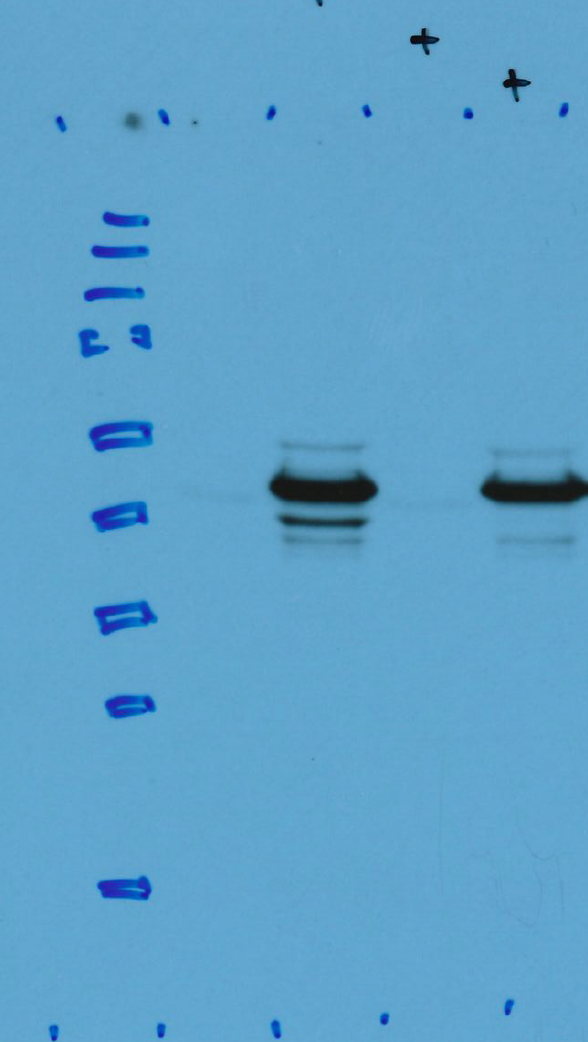

Supplement: Figure 3—figure supplement 1—source data 2. [file elife-110786-fig3-figsupp1-data2.zip › Fig 3-fs1_SD/Figure 3-fs1A_1_CK1d.tif]

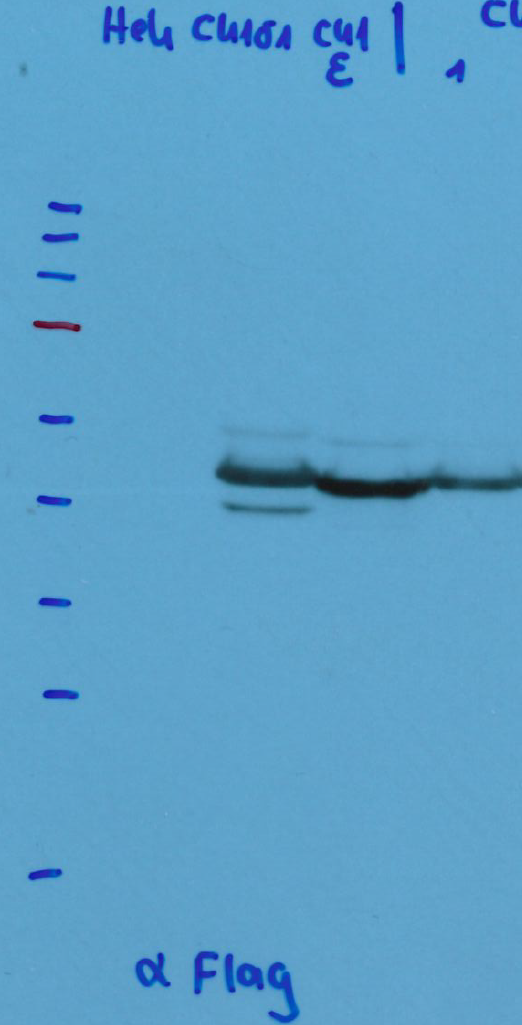

Supplement: Figure 3—figure supplement 1—source data 2. [file elife-110786-fig3-figsupp1-data2.zip › Fig 3-fs1_SD/Figure 3-fs1A_2_FLAG.tif]

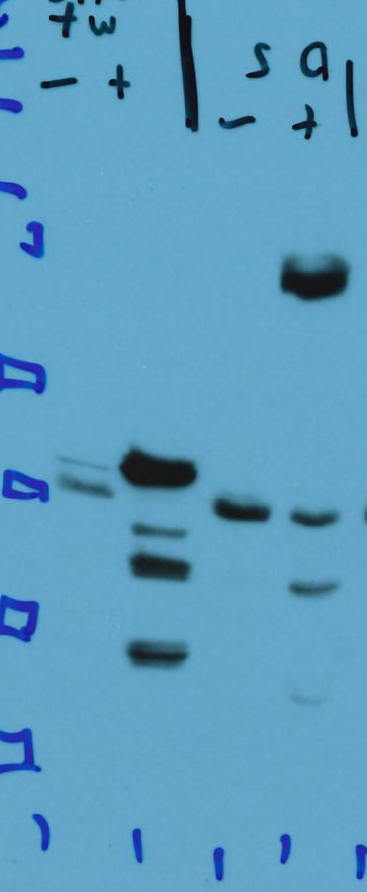

Supplement: Figure 3—figure supplement 1—source data 2. [file elife-110786-fig3-figsupp1-data2.zip › Fig 3-fs1_SD/Figure 3-fs1B,C_CK1d.tif]

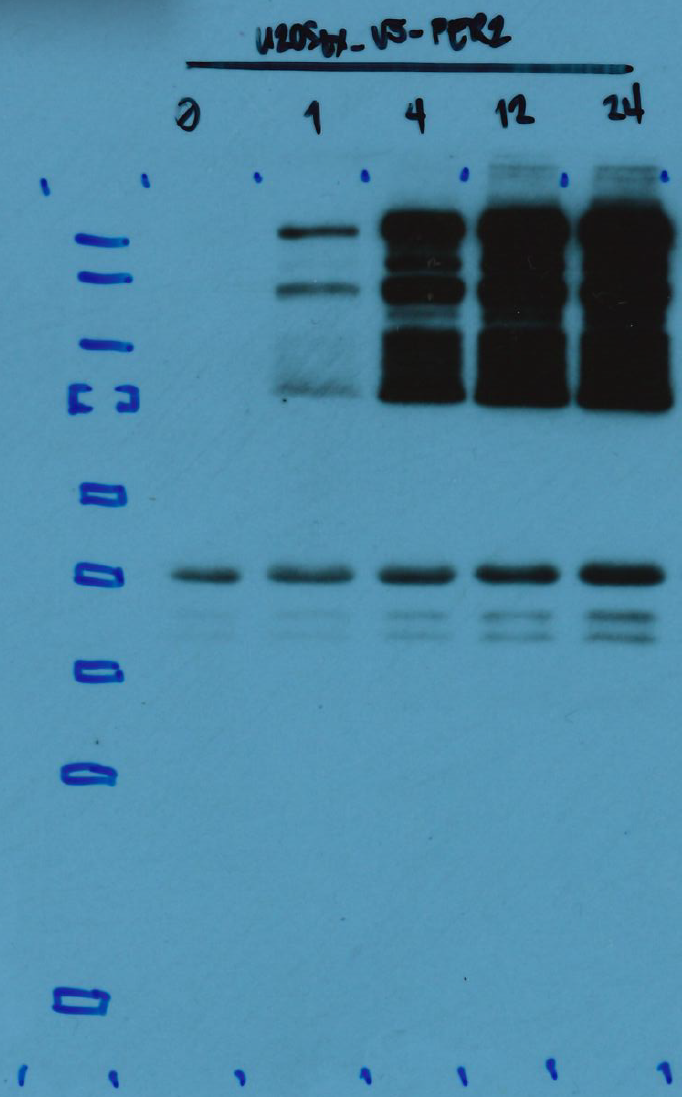

Supplement: Figure 4—source data 2. [file elife-110786-fig4-data2.zip › Fig 4_SD/Figure 4A_longexp_CK1d.tif]

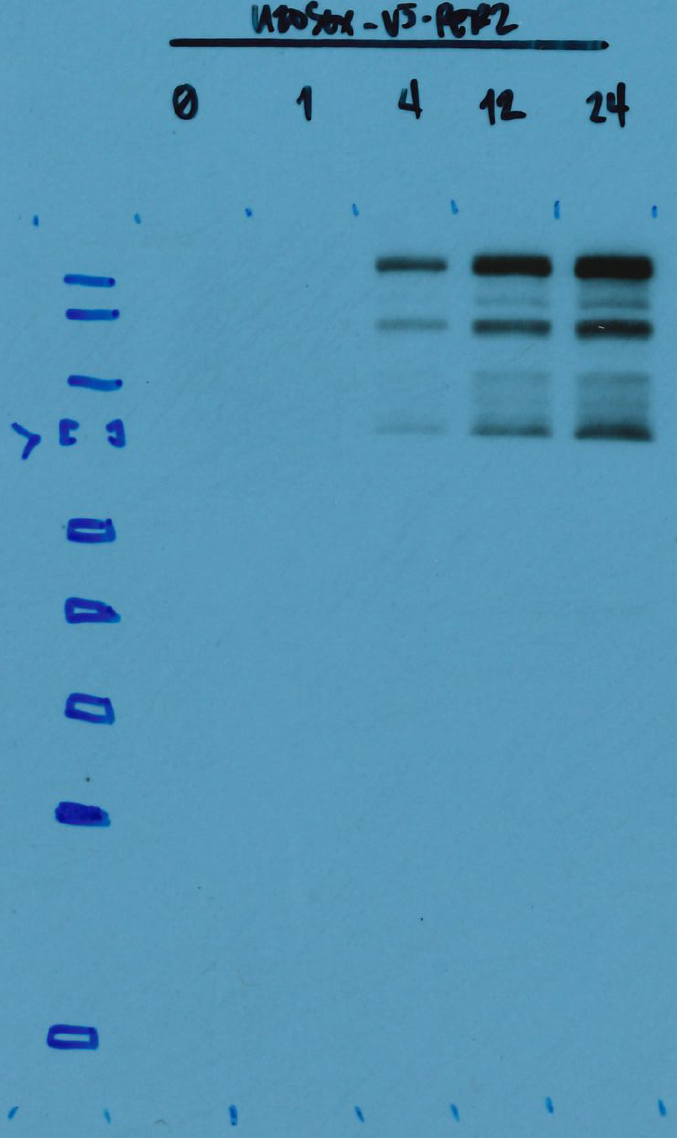

Supplement: Figure 4—source data 2. [file elife-110786-fig4-data2.zip › Fig 4_SD/Figure 4A_shortexp_V5-PER2.tif]

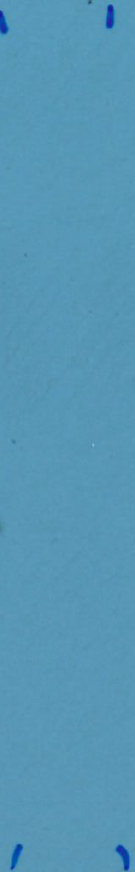

Supplement: Figure 4—source data 2. [file elife-110786-fig4-data2.zip › Fig 4_SD/Figure 4B_PER2dCKBD_1_none.tif]

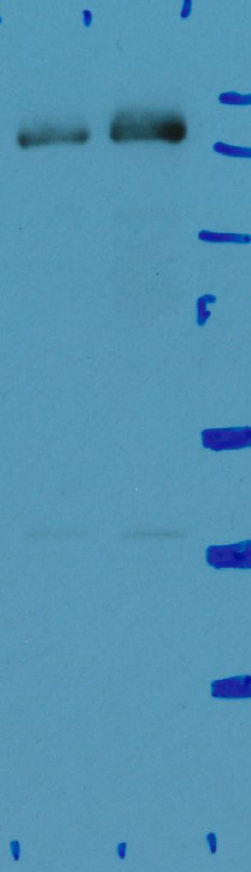

Supplement: Figure 4—source data 2. [file elife-110786-fig4-data2.zip › Fig 4_SD/Figure 4B_PER2dCKBD_2_high.tif]

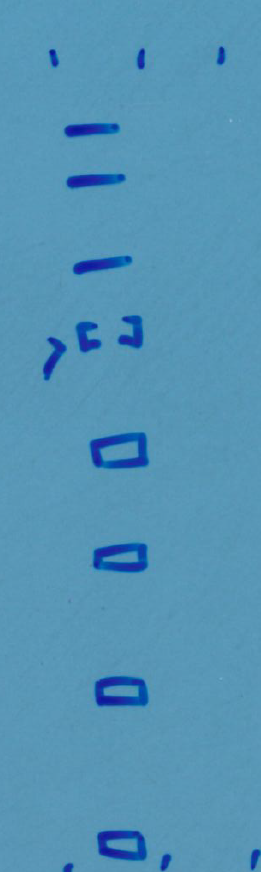

Supplement: Figure 4—source data 2. [file elife-110786-fig4-data2.zip › Fig 4_SD/Figure 4B_PER2_1_none.tif]

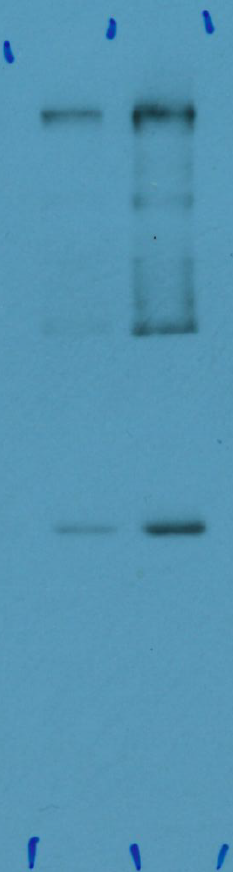

Supplement: Figure 4—source data 2. [file elife-110786-fig4-data2.zip › Fig 4_SD/Figure 4B_PER2_2_high.tif]

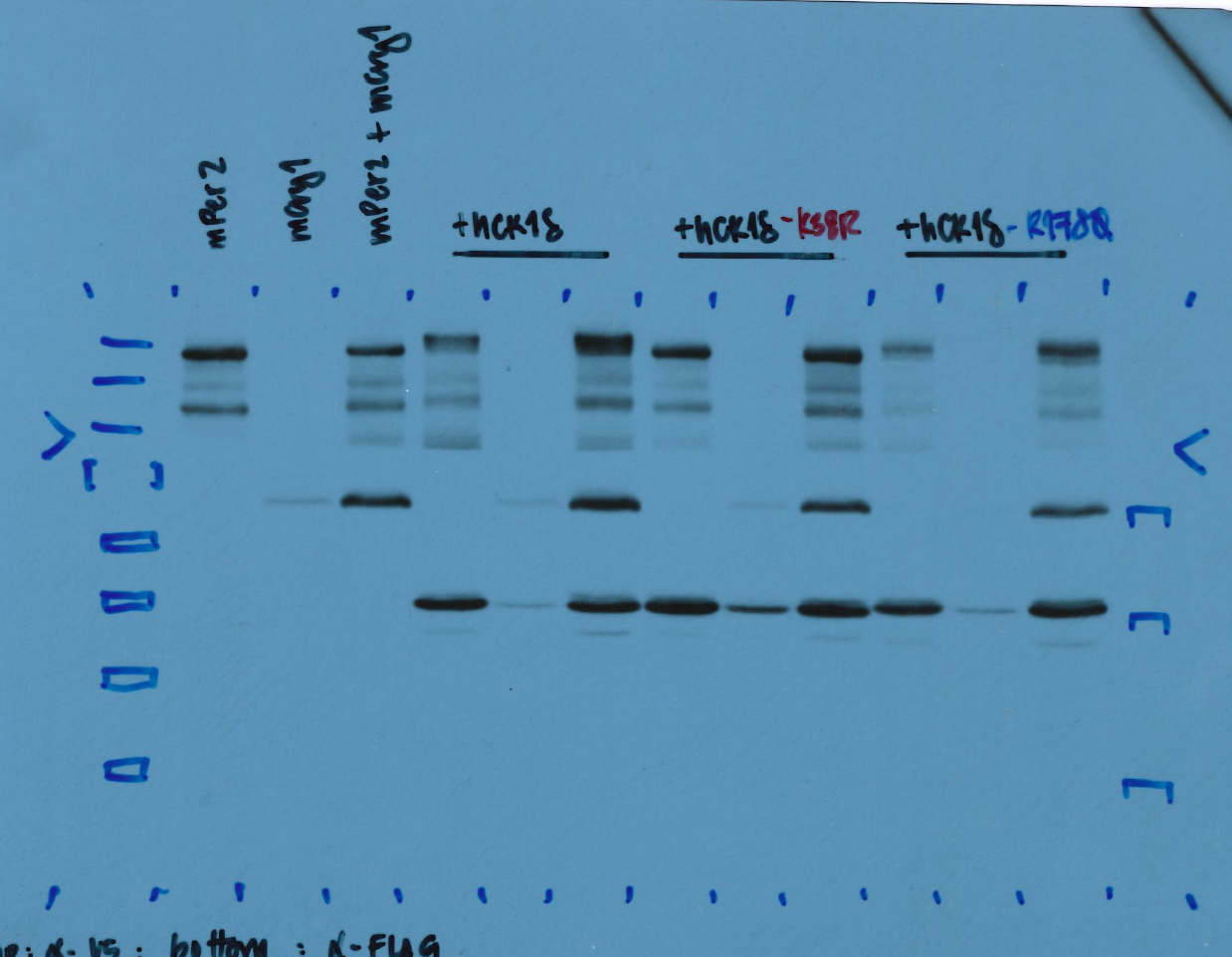

Supplement: Figure 4—source data 2. [file elife-110786-fig4-data2.zip › Fig 4_SD/Figure 4C_V5_FLAG.tif]

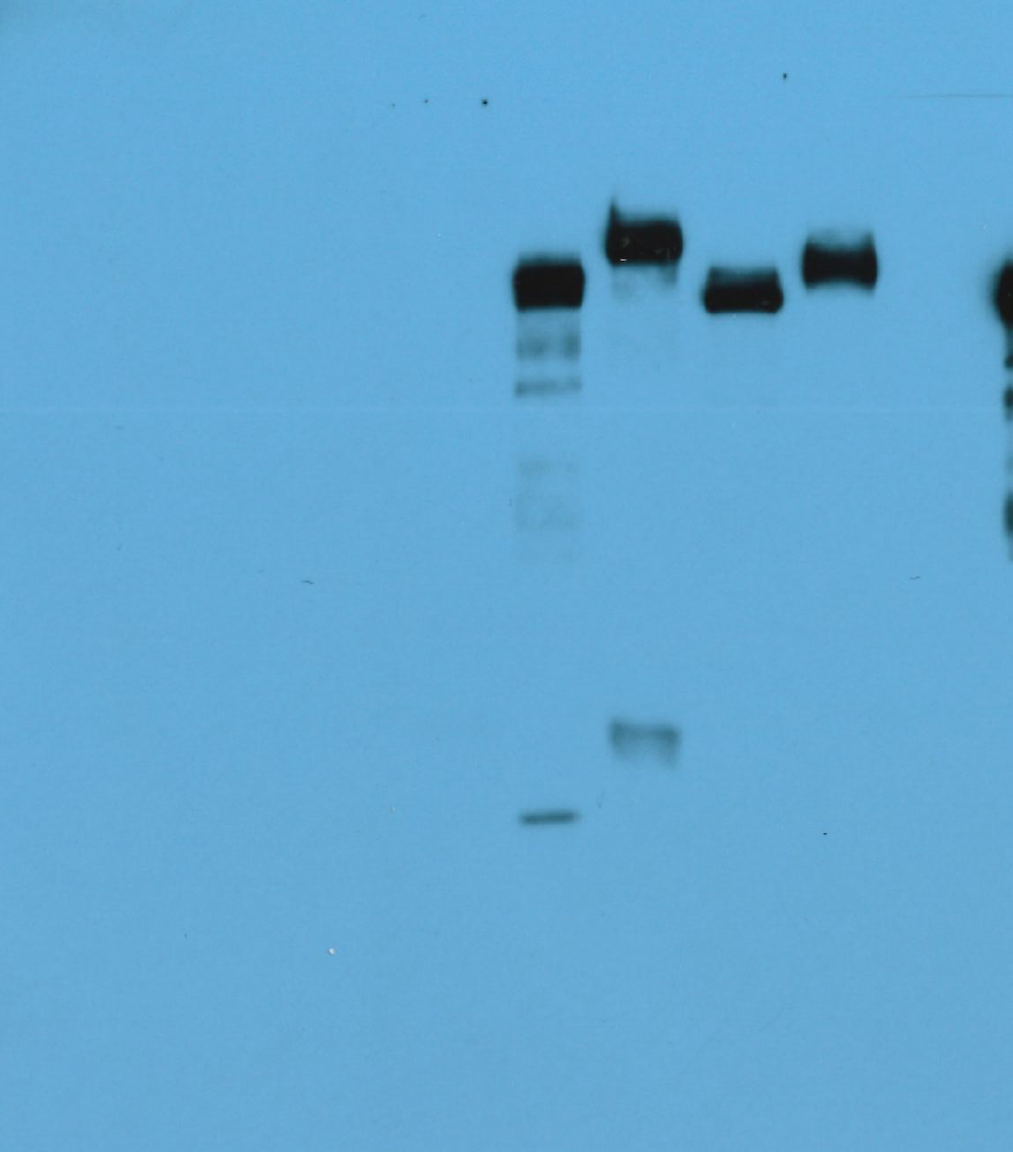

Supplement: Figure 4—source data 2. [file elife-110786-fig4-data2.zip › Fig 4_SD/Figure 4D_1_V5_PER2.tif]

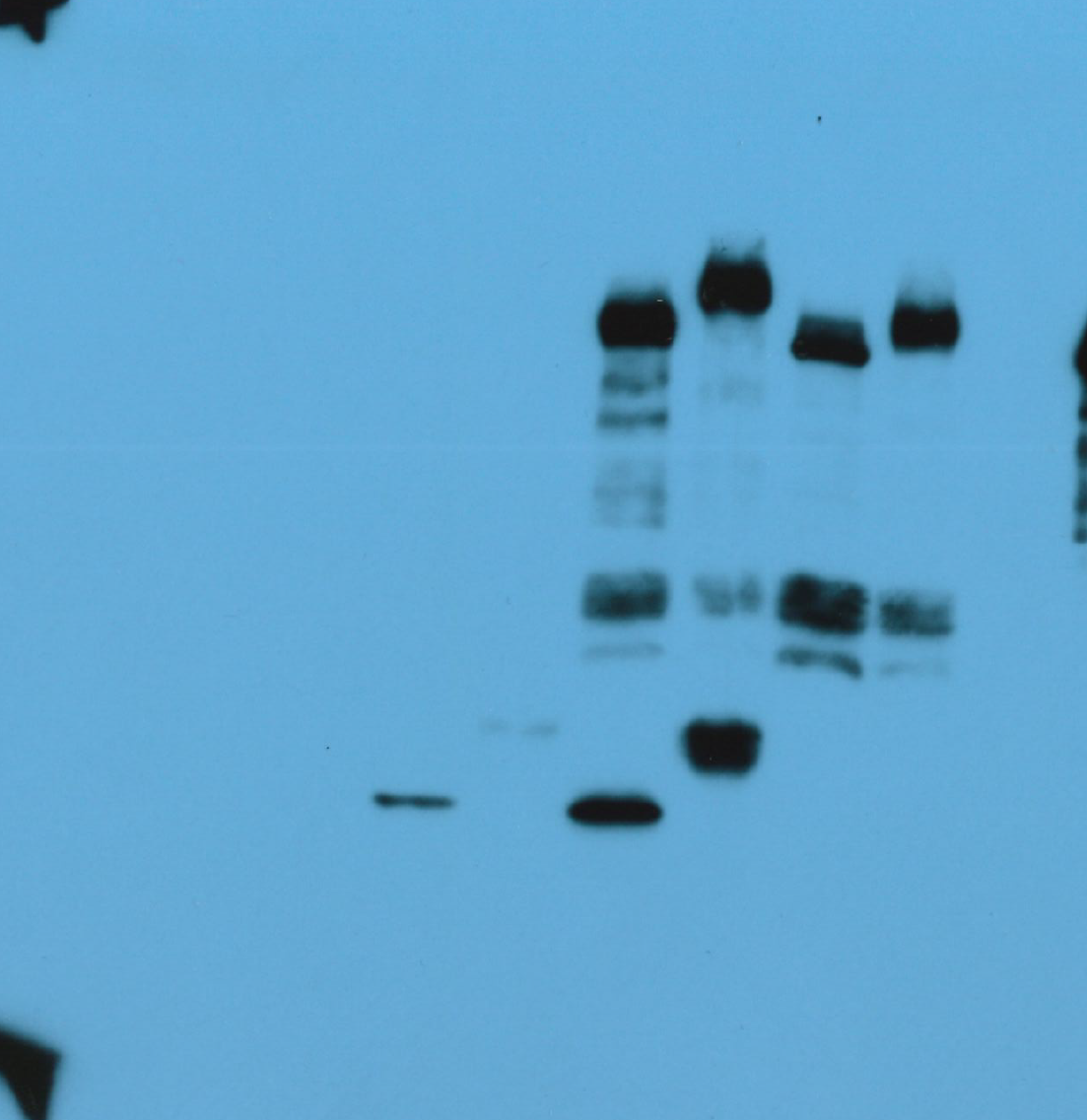

Supplement: Figure 4—source data 2. [file elife-110786-fig4-data2.zip › Fig 4_SD/Figure 4D_2_FLAG_CRY1.tif]

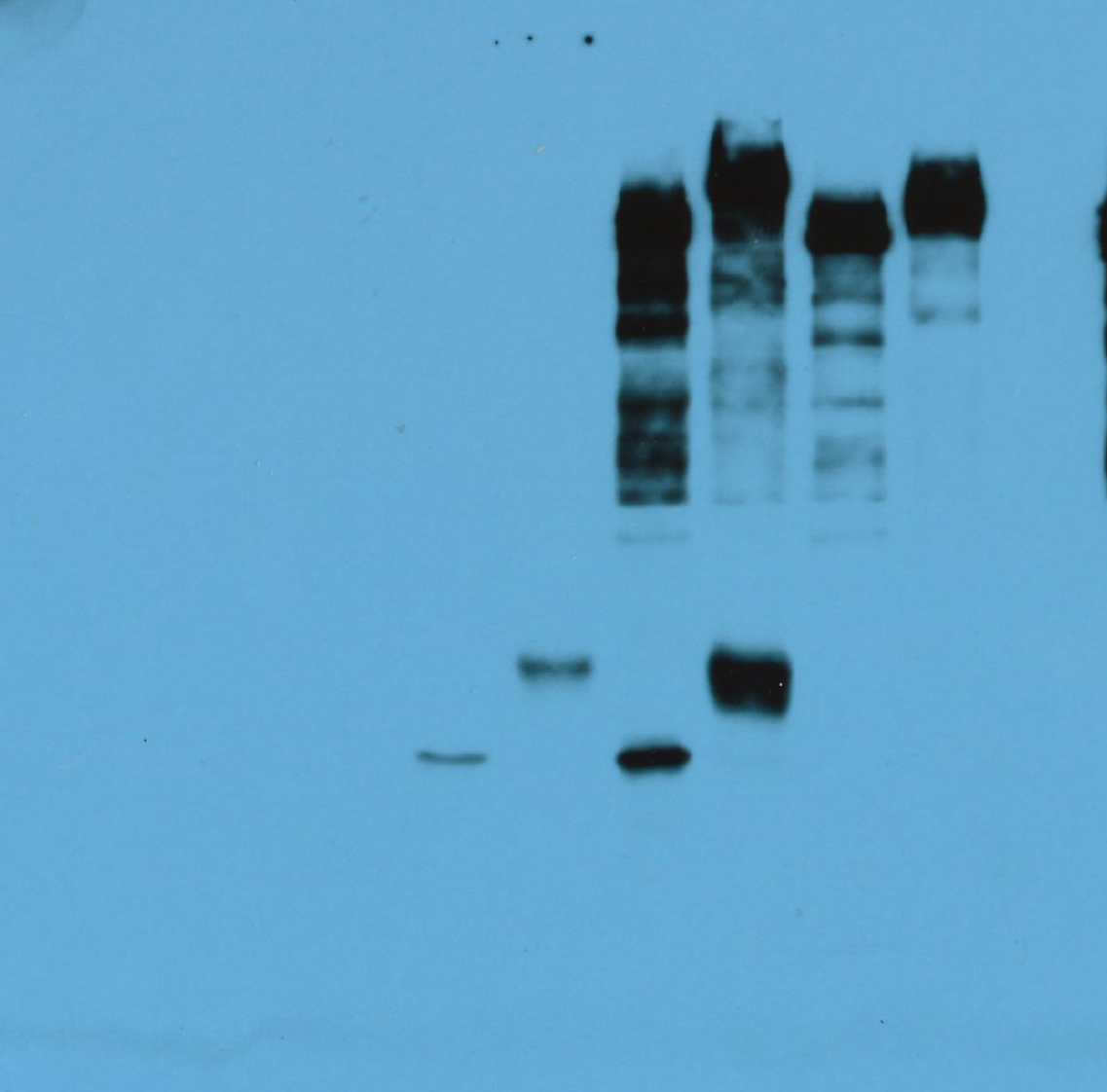

Supplement: Figure 4—source data 2. [file elife-110786-fig4-data2.zip › Fig 4_SD/Figure 4D_3_CK1d_shortexp.tif]

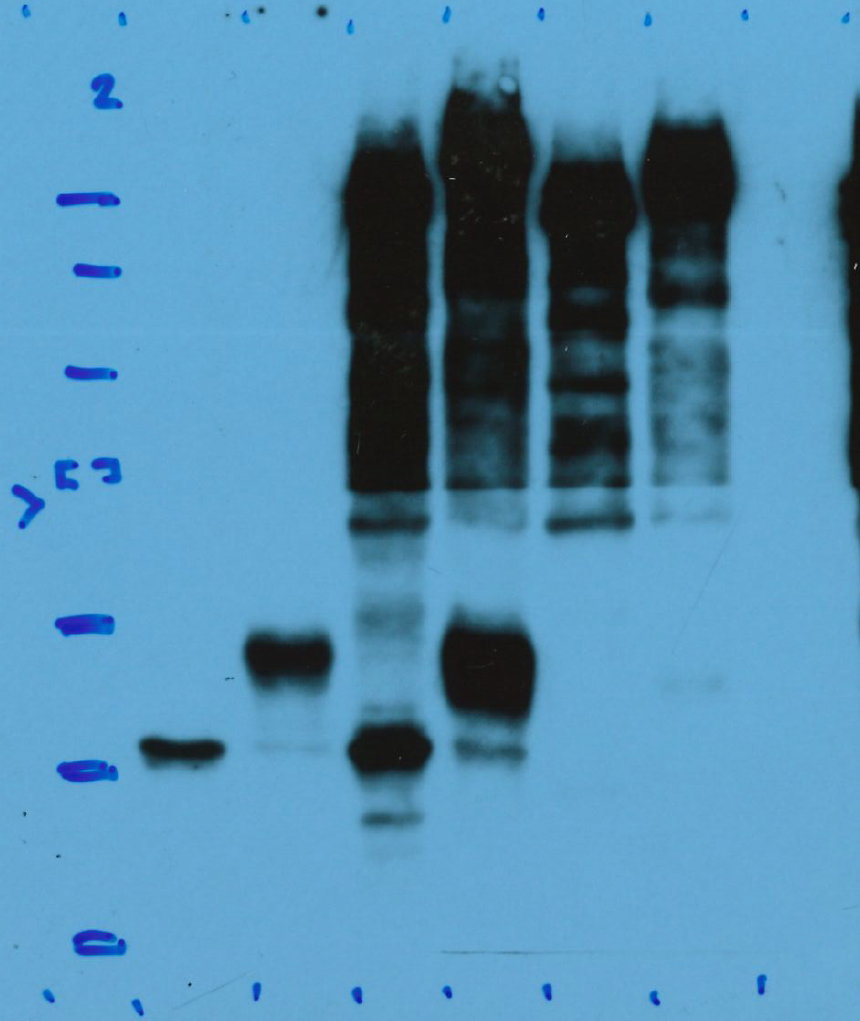

Supplement: Figure 4—source data 2. [file elife-110786-fig4-data2.zip › Fig 4_SD/Figure 4D_4_CK1d_longexp.tif]

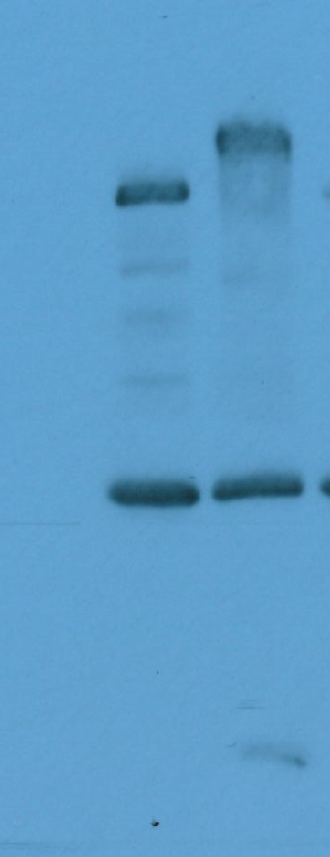

Supplement: Figure 6—source data 2. [file elife-110786-fig6-data2.zip › Fig 6_SD/Figure 6C_1-Load.tif]

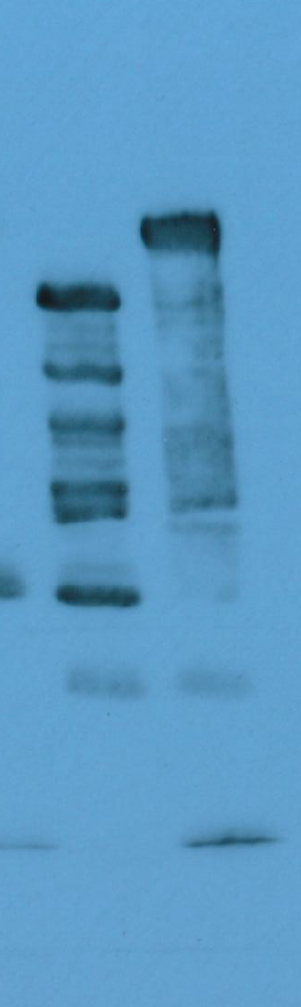

Supplement: Figure 6—source data 2. [file elife-110786-fig6-data2.zip › Fig 6_SD/Figure 6C_2-IP.tif]

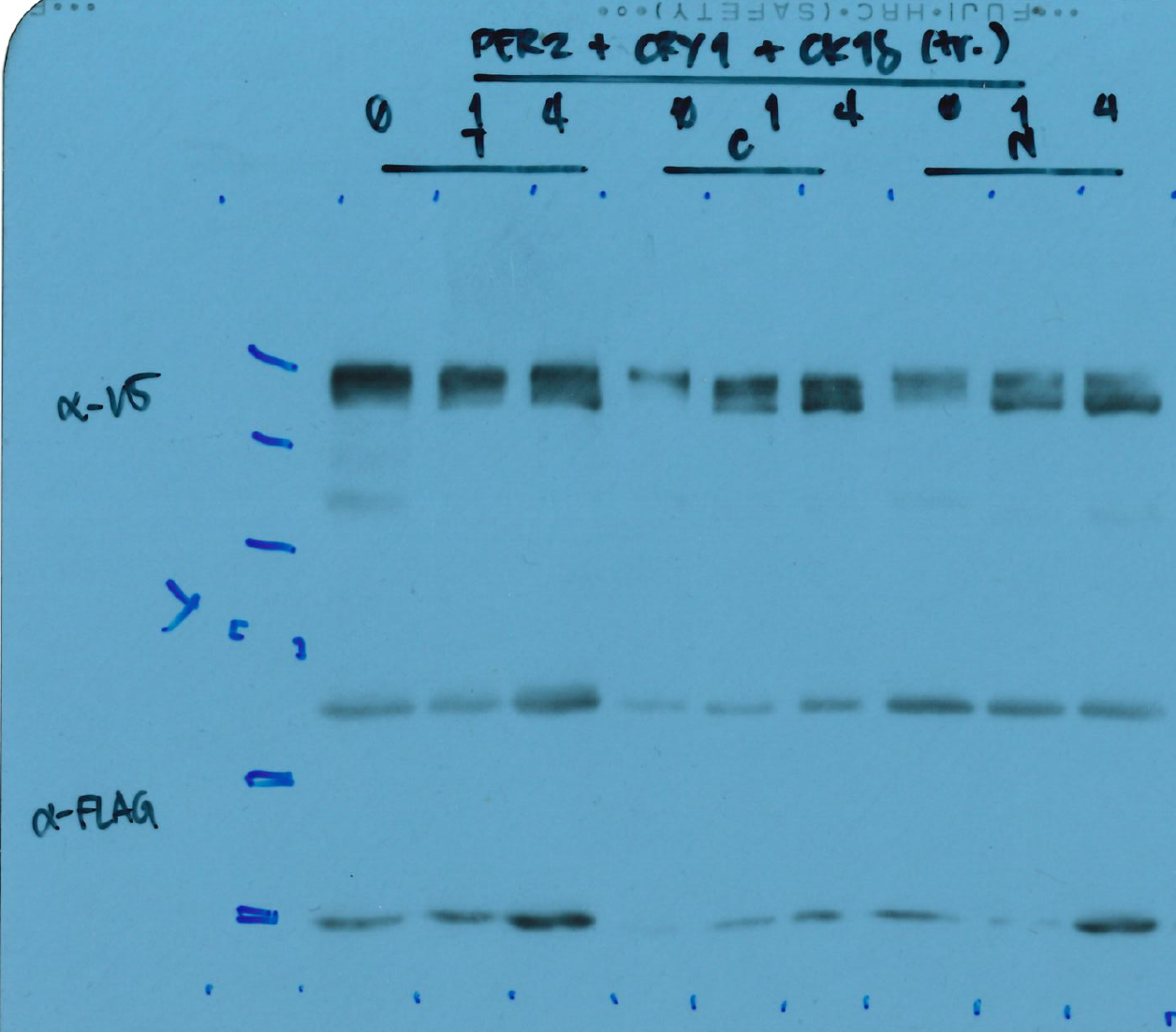

Supplement: Figure 6—source data 2. [file elife-110786-fig6-data2.zip › Fig 6_SD/Figure 6E_V5-PER2.tif]

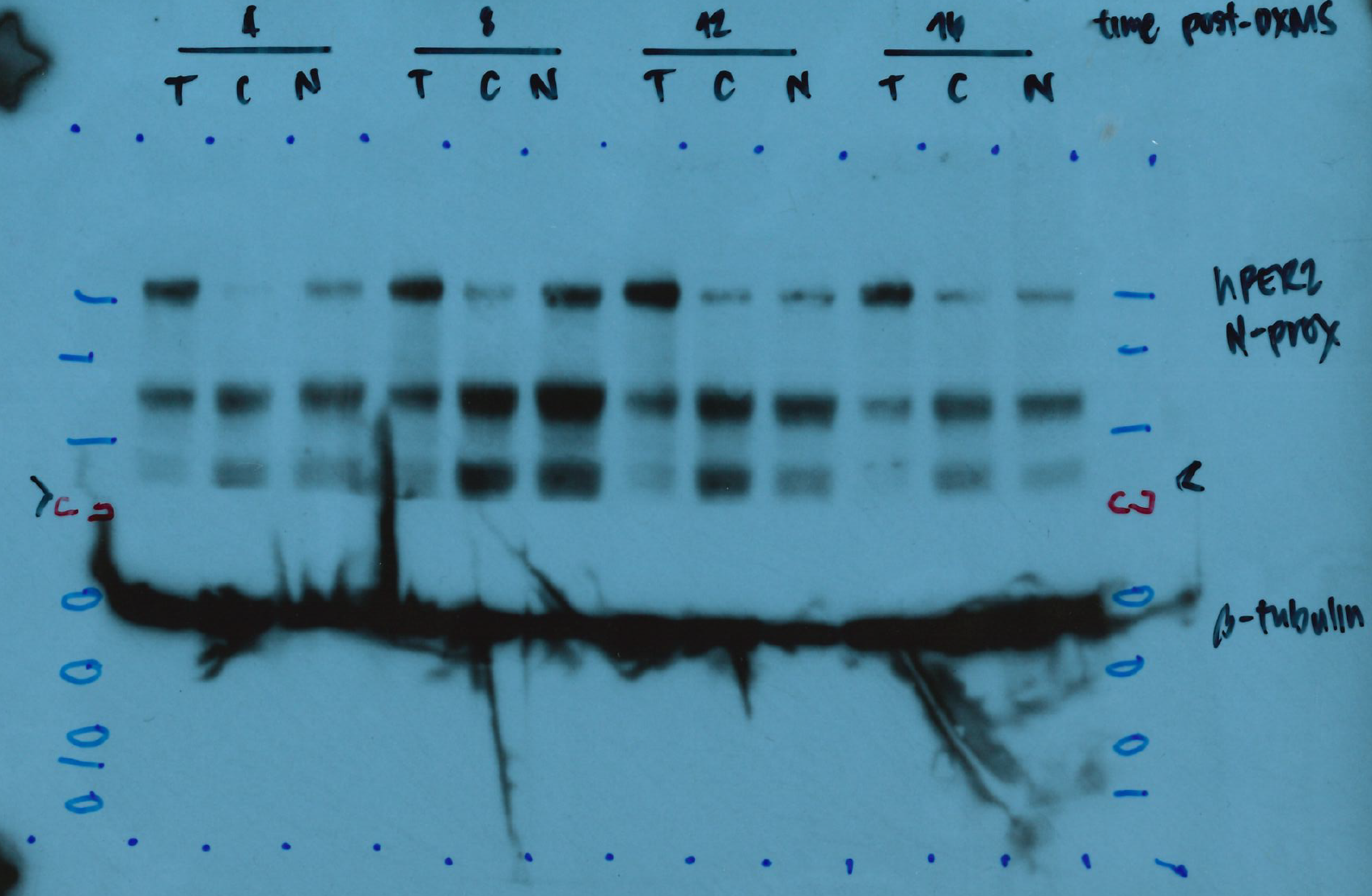

Supplement: Figure 6—source data 2. [file elife-110786-fig6-data2.zip › Fig 6_SD/Figure 6F_hPER2.tif]

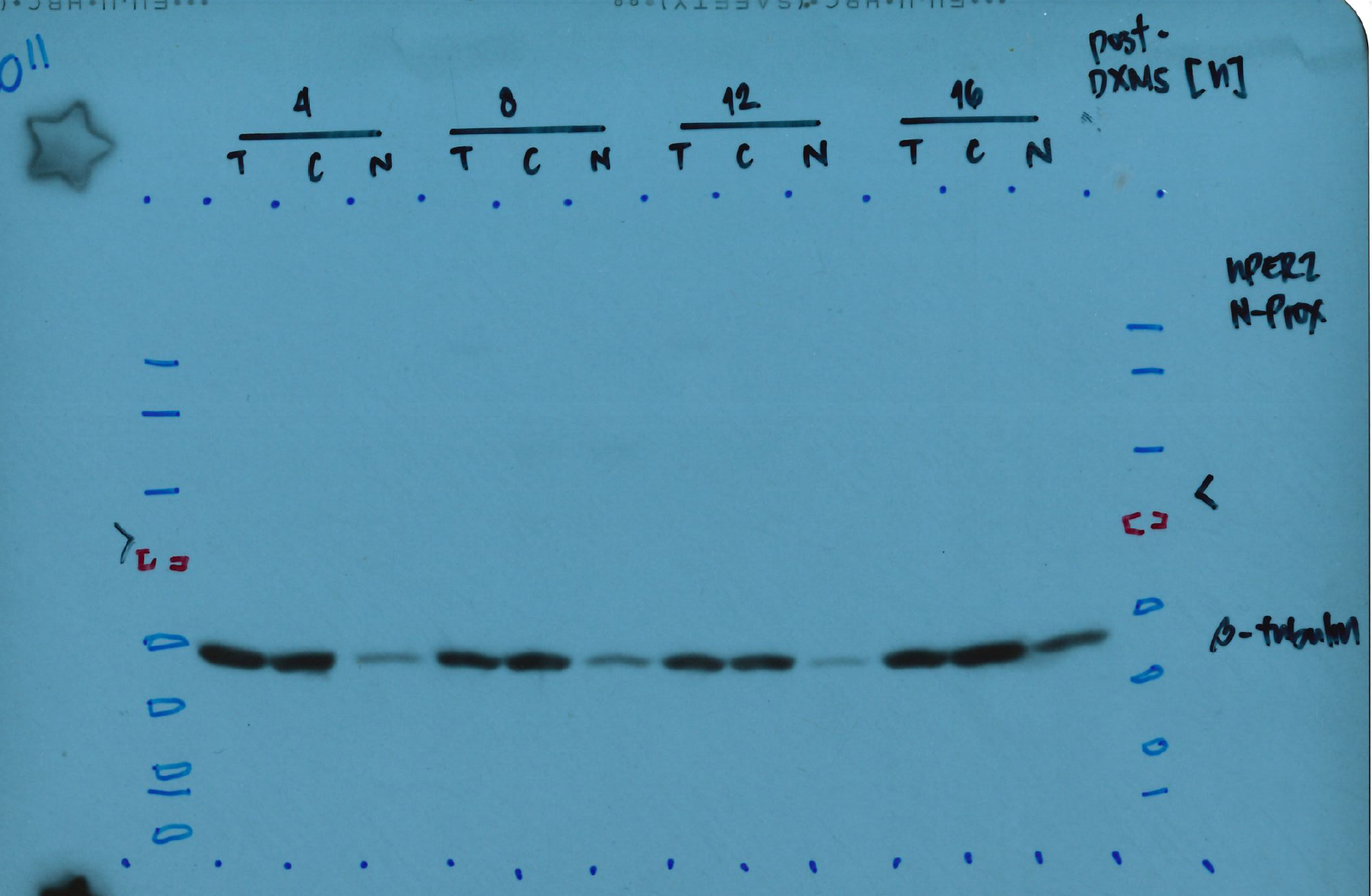

Supplement: Figure 6—figure supplement 1—source data 2. [file elife-110786-fig6-figsupp1-data2.zip › Fig 6-fs1_SD/Figure 6-fs1D_beta-TUB.tif]

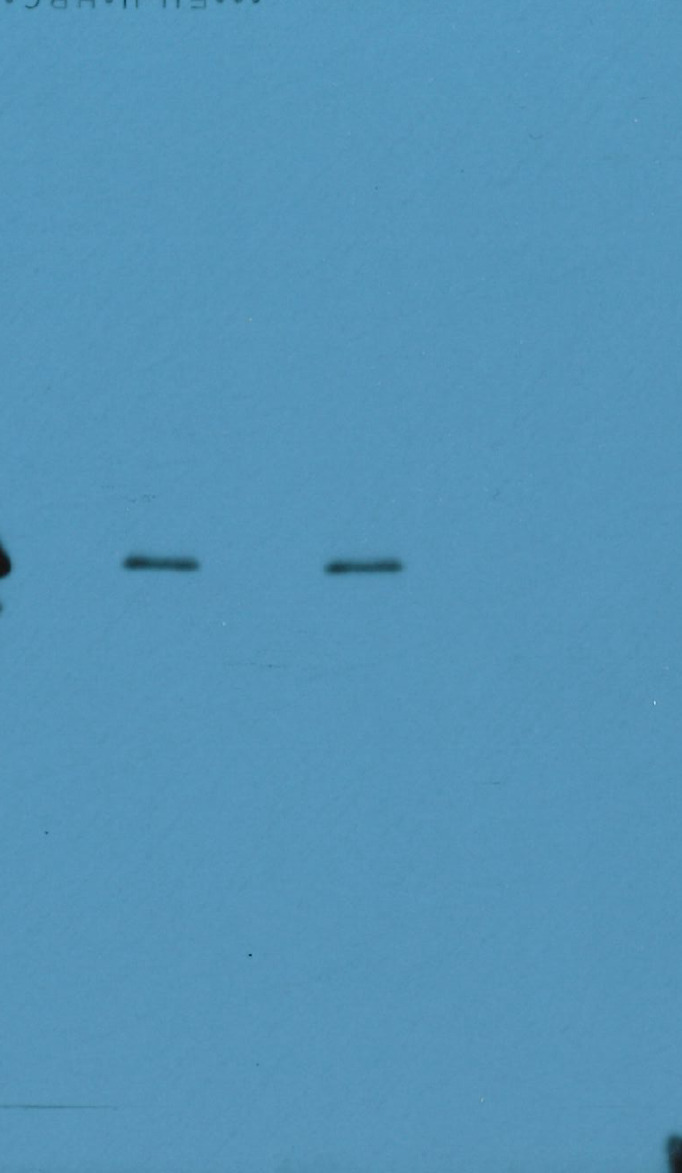

Supplement: Figure 7—source data 2. [file elife-110786-fig7-data2.zip › Fig 7_SD/Figure 7D_V5.tif]
