# Supplementary material for: Nuclear CK1δ as a critical determinant of PER:CRY complex dynamics and circadian period
Source: eLife. 2026 Jun 15;15:RP110786. doi: 10.7554/eLife.110786 (PMC13268647; doi:10.7554/eLife.110786)
Supplement: Figure 3—source data 1. [file elife-110786-fig3-data1.docx]

**
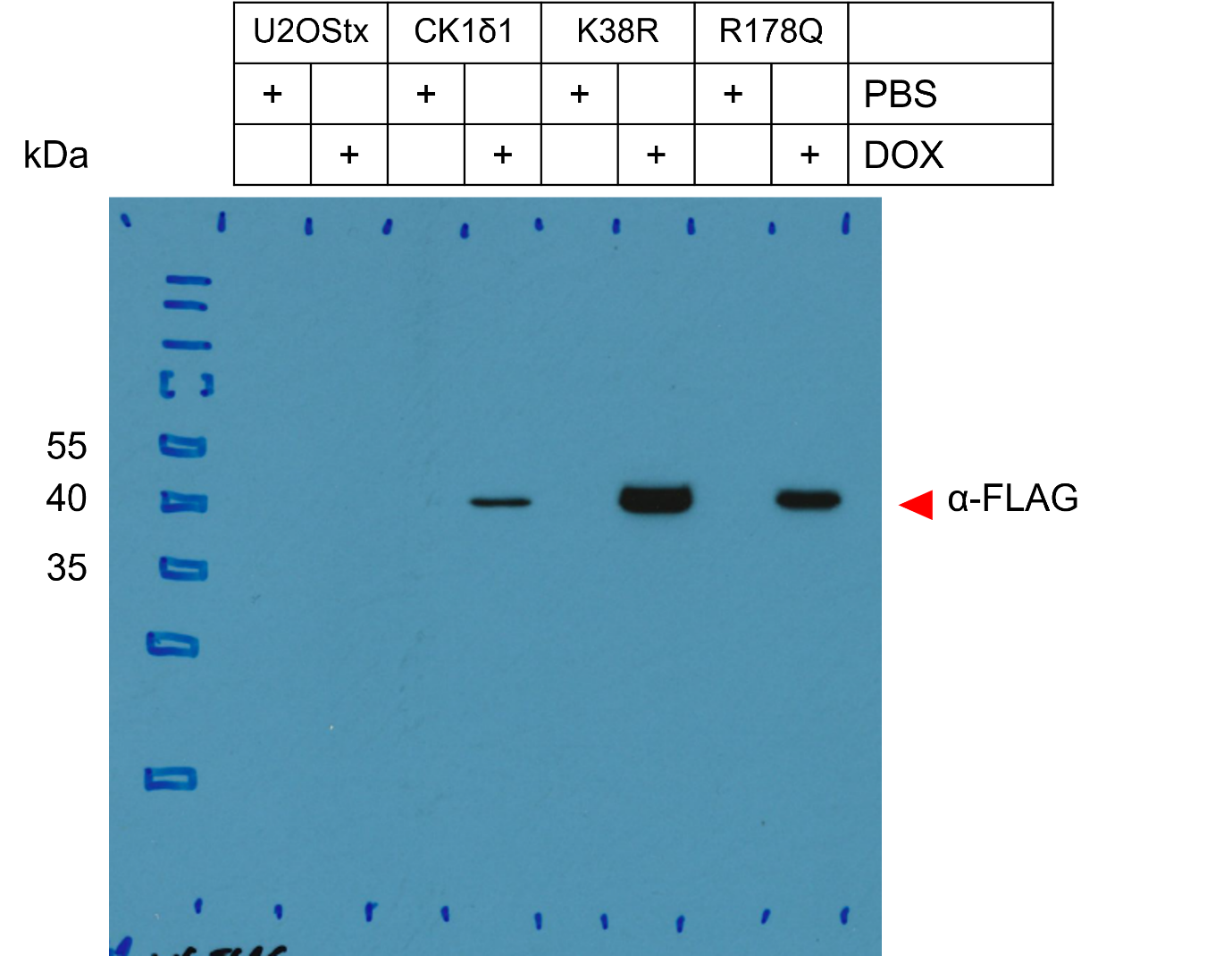
**

**Figure 3B – Source Data 1a.** Original film corresponding to Figure 3B, upper panel. Using the basal U2OStx line and the stable cell line generated to induce either wild-type CK1δ1, kinase-dead CK1δ1 (CK1δ1-K38R), and tau-like CK1δ1 (CK1δ1-R178Q), cells were treated with either PBS (negative control) or DOX (doxycycline) to induce protein expression. Resulting blots were decorated with anti-FLAG antibody to detect overexpressed CK1δ1.


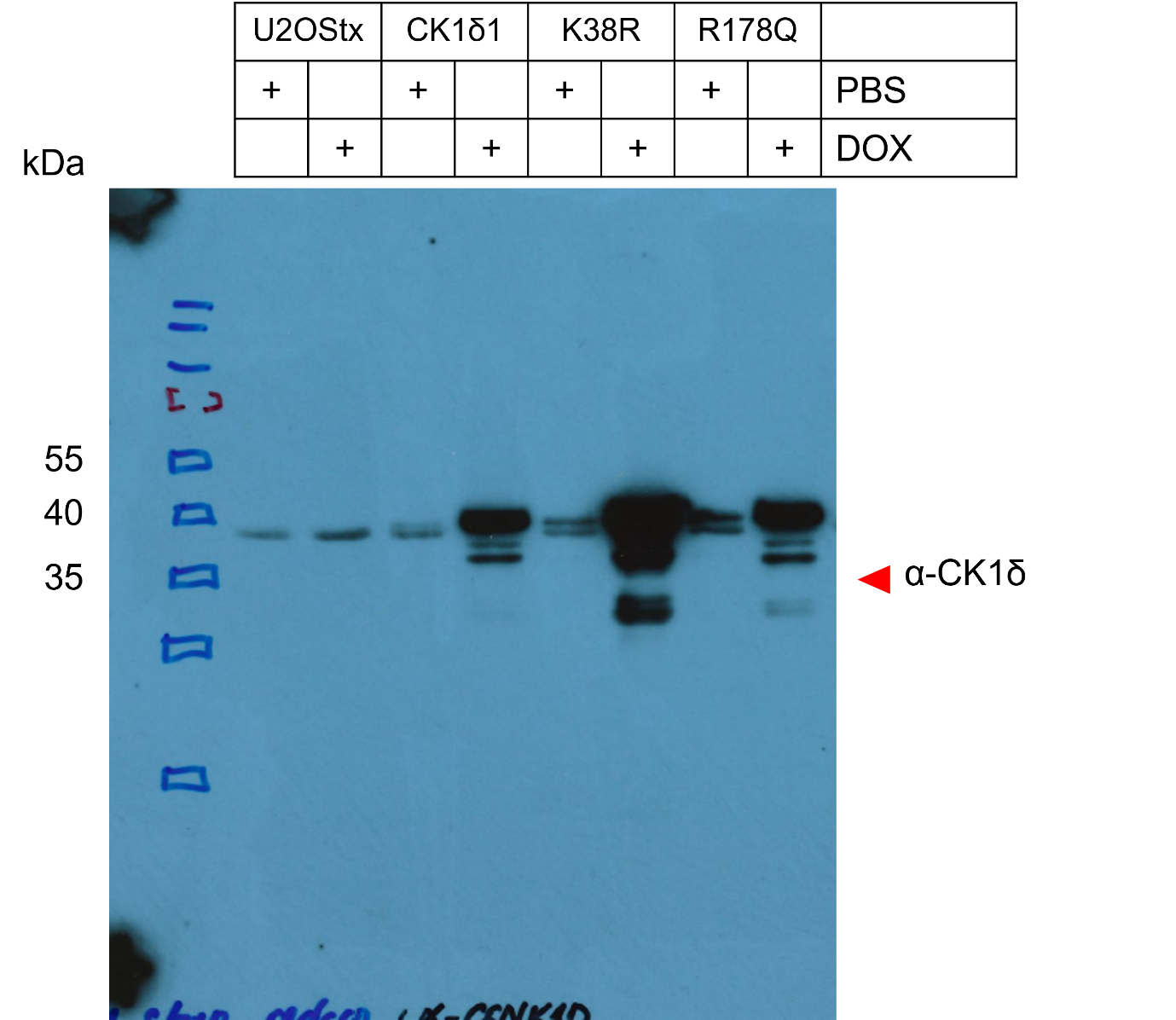


**Figure 3B – Source Data 1b.** Original film corresponding to Figure 3B, lower panel. Using the basal U2OStx line and the stable cell line generated to induce either wild-type CK1δ1, kinase-dead CK1δ1 (CK1δ1-K38R), and tau-like CK1δ1 (CK1δ1-R178Q), cells were treated with either PBS (negative control) or DOX (doxycycline) to induce protein expression. Resulting blots were decorated with anti-CK1δ antibody (abcam, ab85320) to detect both endogenous and overexpressed CK1δ1.


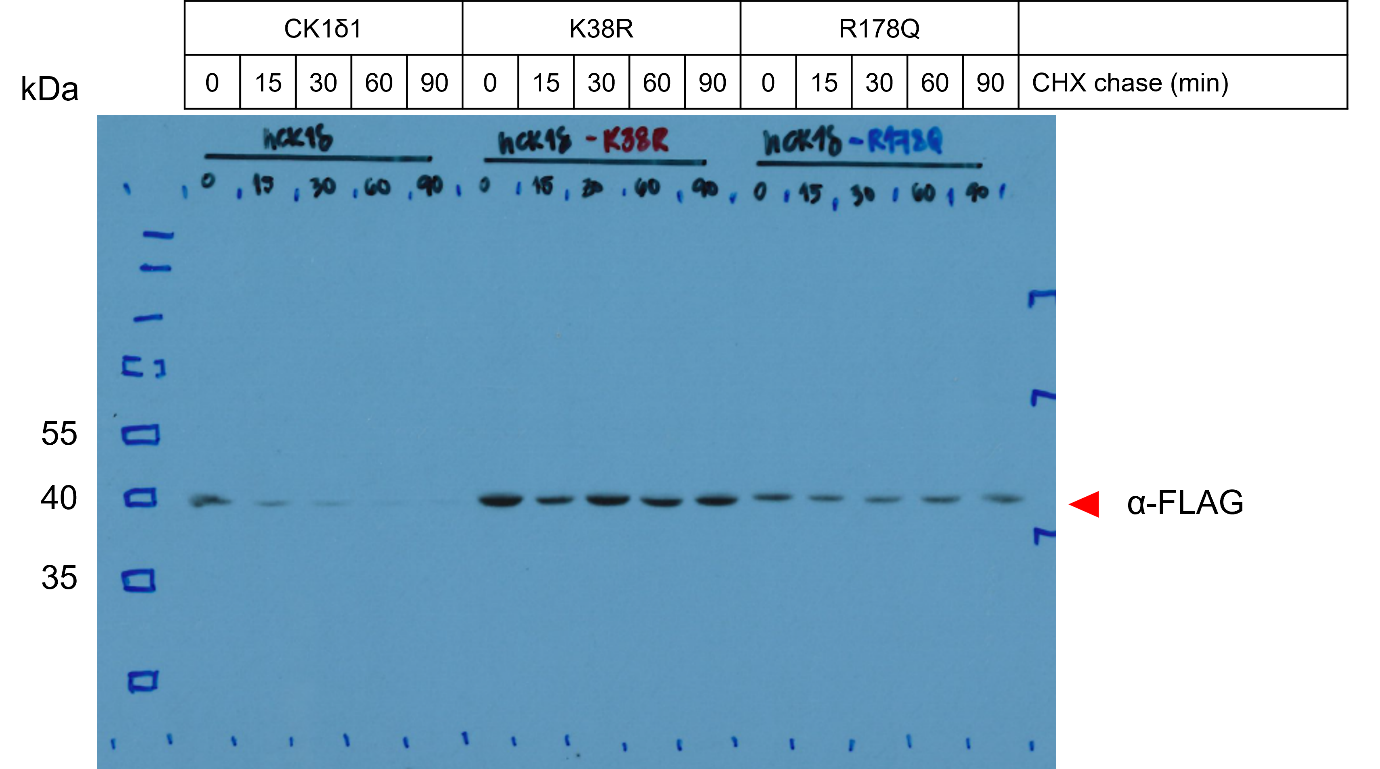


**Figure 3C – Source Data 1.** Original film corresponding to Figure 3C. Wild-type CK1δ1, kinase-dead CK1δ1 (CK1δ1-K38R), and tau-like CK1δ1 (CK1δ1-R178Q) were induced and treated with CHX to arrest protein translation and assess the stability of the kinase. Resulting blots were decorated with anti-FLAG antibody.


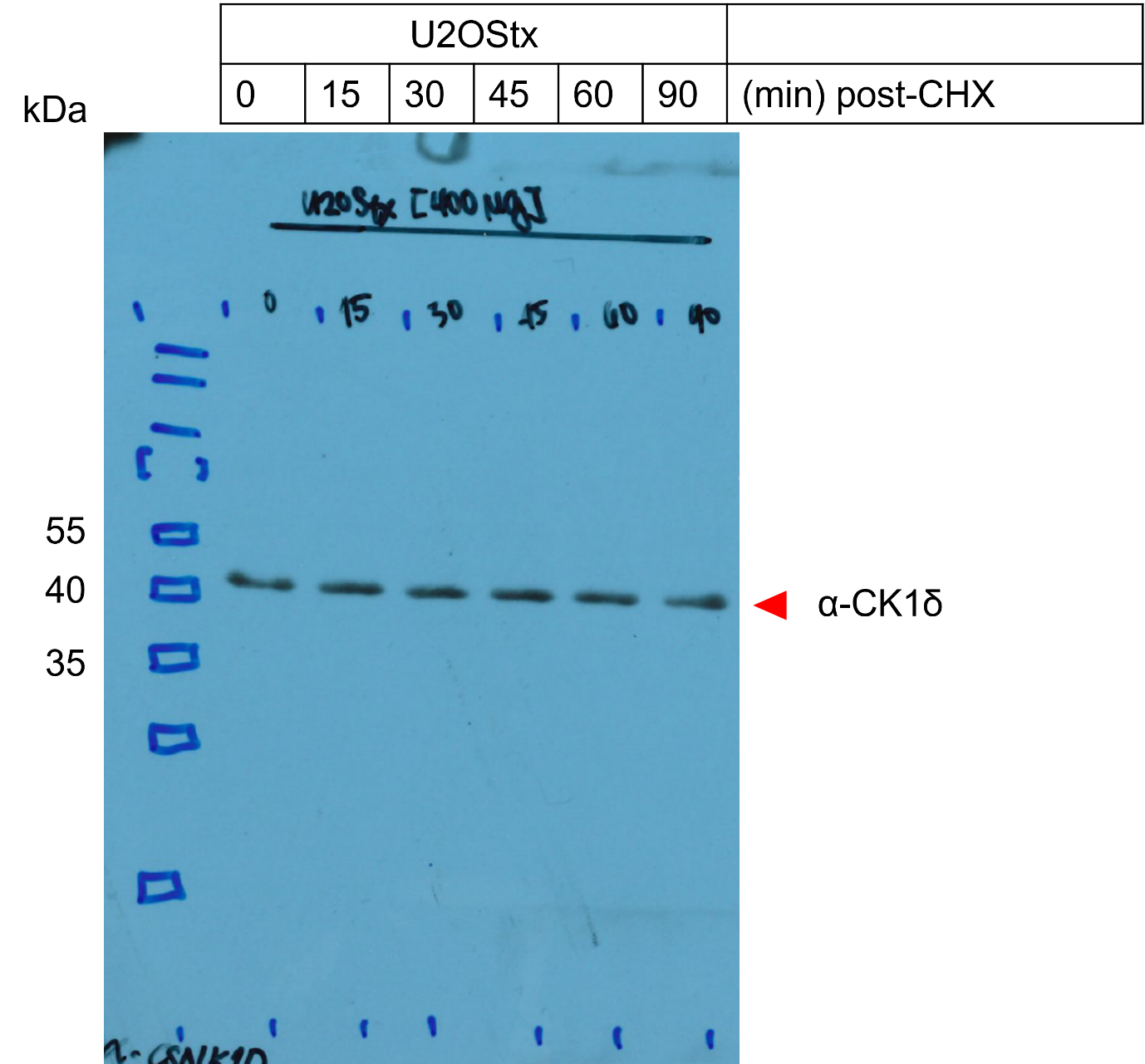


**Figure 3D – Source Data 1.** Original film corresponding to Figure 3D. U2OStx cells were treated with CHX and protein samples were for immunoblotting. The blot was decorated with anti-CK1δ antibody.


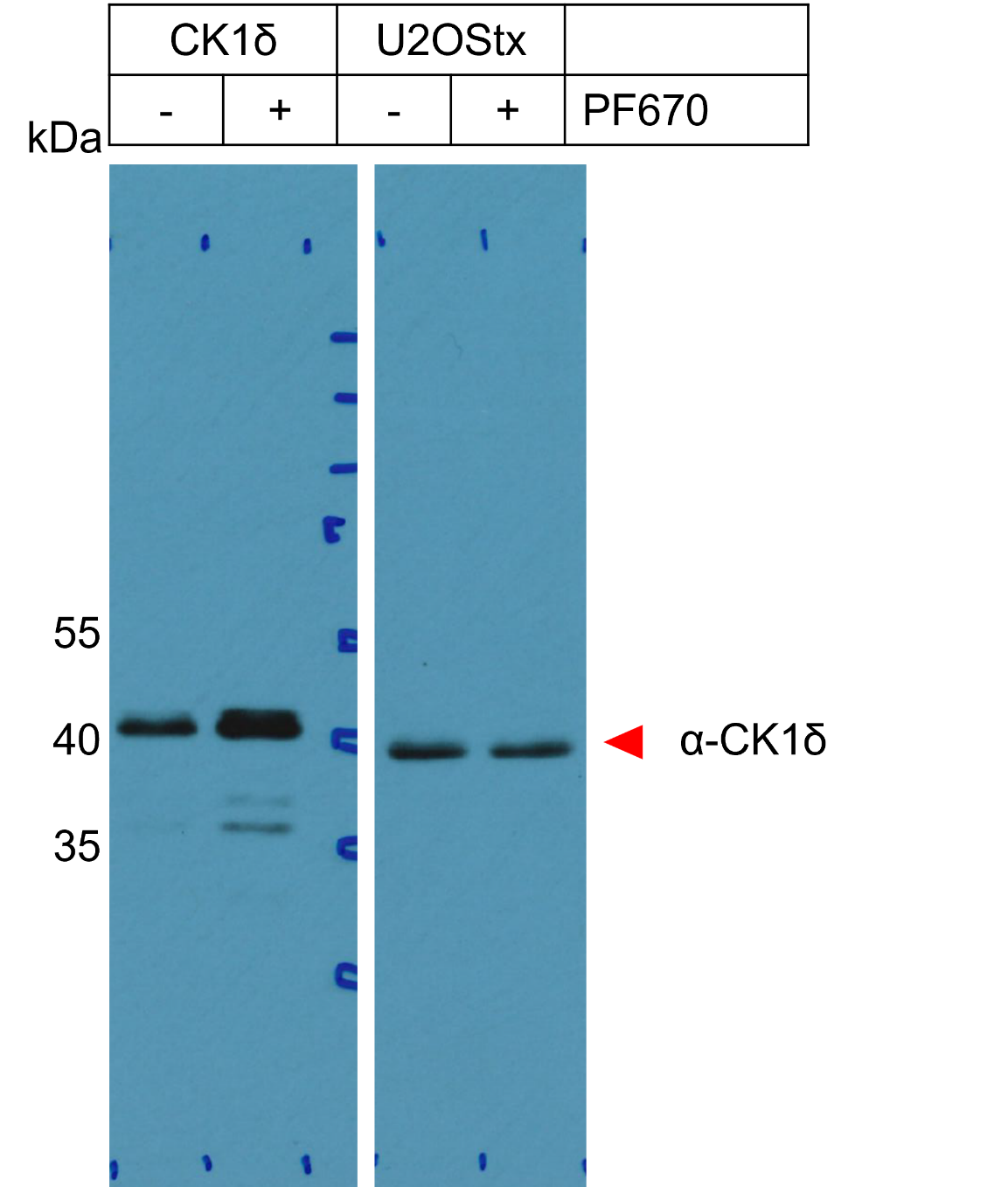


**Figure 3F – Source Data 1.** Original film corresponding to Figure 3F. U2OStx and U2OStx_CK1δ were treated with PF670 and protein samples were extracted to assess CK1δ stability. Resulting blots were decorated with anti-CK1δ antibody.


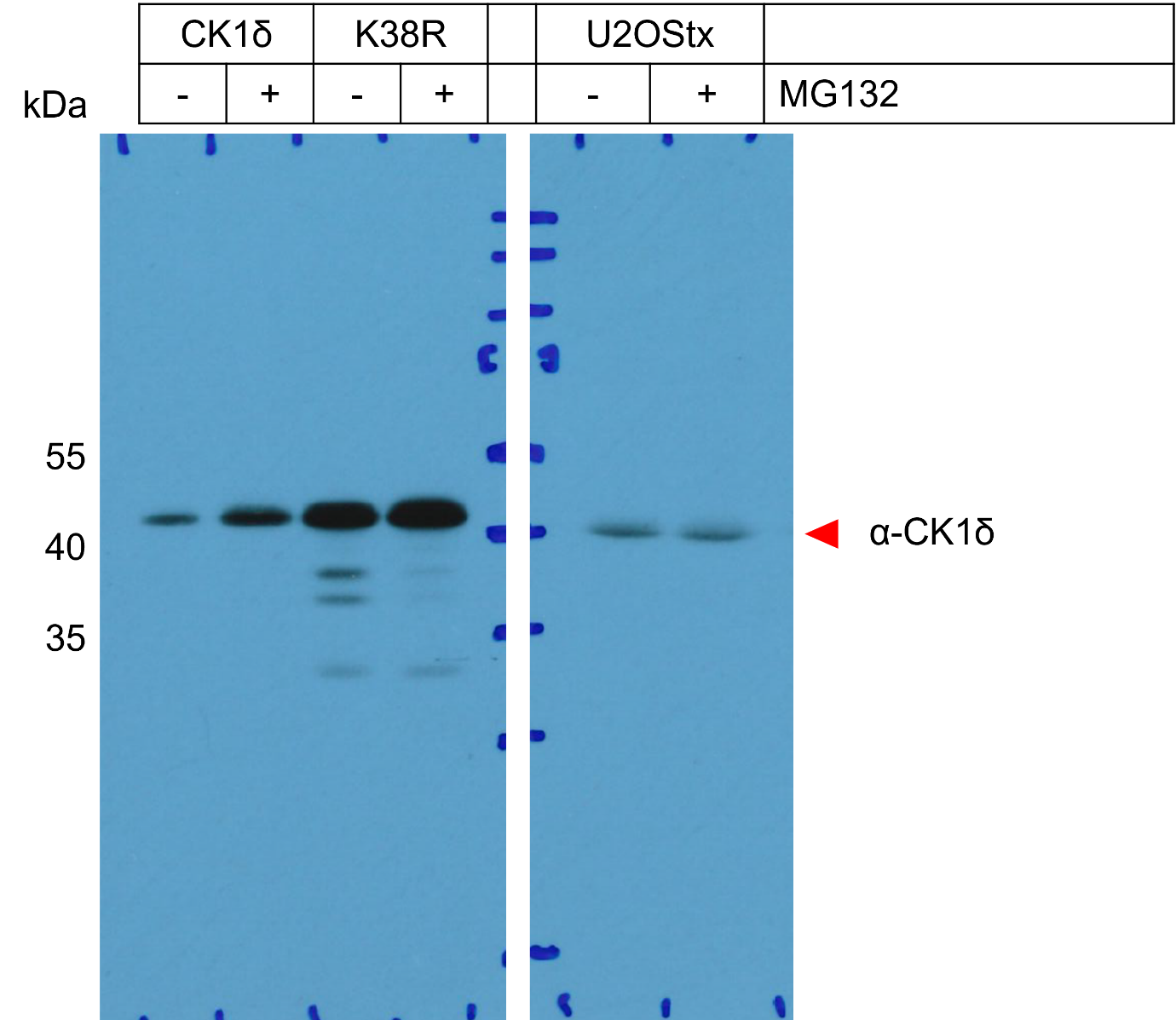


**Figure 3G – Source Data 1.** Original film corresponding to Figure 3G. U2OStx, U2OStx_CK1δ and U2OStx_CK1δ-K38R cells were treated with MG132 and protein samples were extracted for immunoblotting. The blot was decorated with anti-CK1δ antibody.
