# Supplementary material for: Nuclear CK1δ as a critical determinant of PER:CRY complex dynamics and circadian period
Source: eLife. 2026 Jun 15;15:RP110786. doi: 10.7554/eLife.110786 (PMC13268647; doi:10.7554/eLife.110786)
Supplement: Figure 3—figure supplement 1—source data 1. [file elife-110786-fig3-figsupp1-data1.docx]

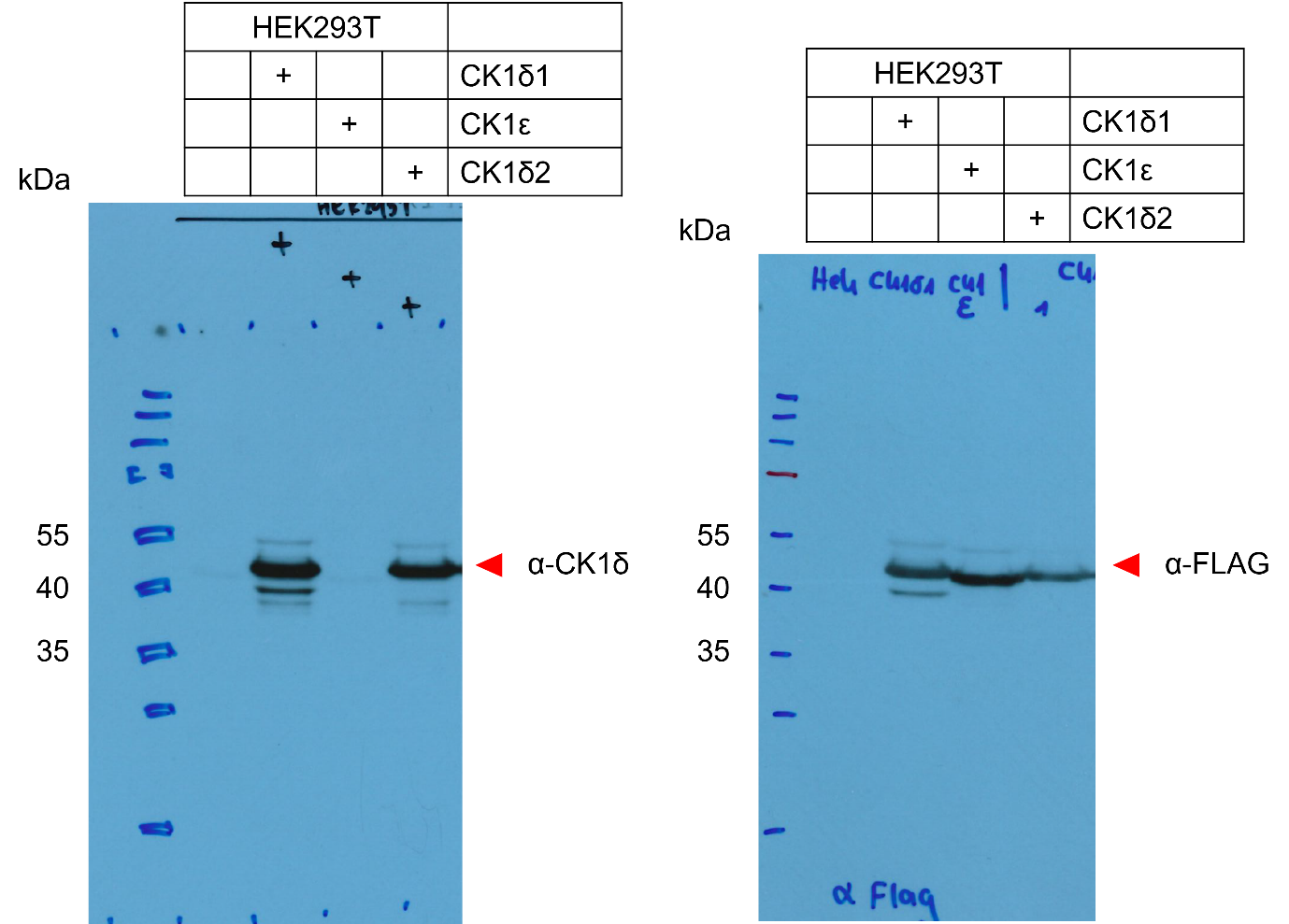


**Figure 3 – figure supplement 1A – Source Data 1.** Original film corresponding to Figure 3 – figure supplement 1A. HEK293T cells were transfected with either CK1δ1, CK1ε, or CK1δ2 and protein samples were extracted for immunoblotting. The blot was decorated with anti-CK1δ antibody, then stripped and redecorated with anti-FLAG antibody.


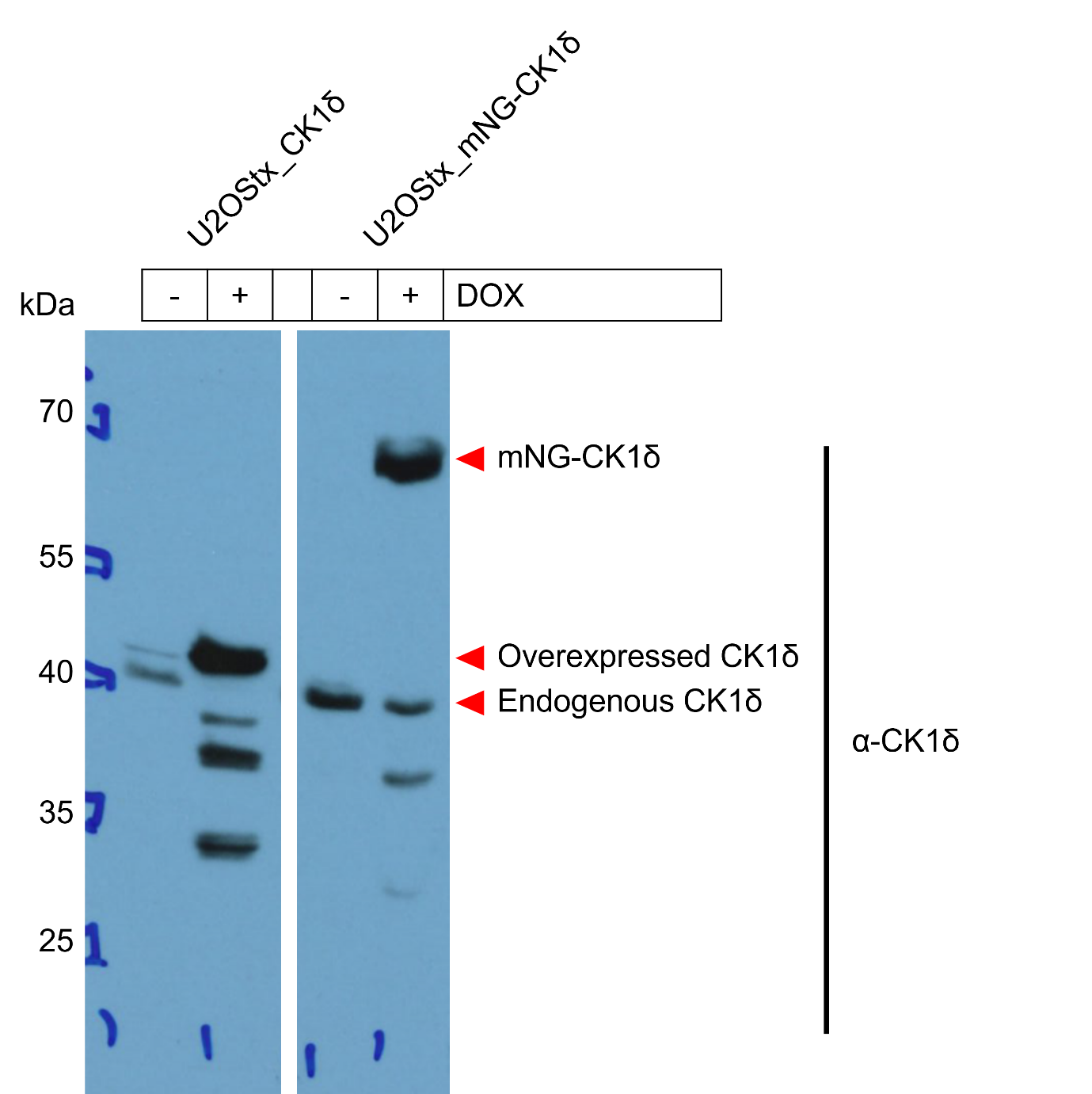


**Figure 3 – figure supplement 1B, C – Source Data 1.** Original film corresponding to Figure 3 . figure supplement 1 – panels B and C. Stable U2OStx_CK1δ and U2OStx_mNG-CK1δ cells were induced with DOX and protein samples were taken for immunoblotting. The blot was decorated with anti-CK1δ antibody.
