# Supplementary material for: Nuclear CK1δ as a critical determinant of PER:CRY complex dynamics and circadian period
Source: eLife. 2026 Jun 15;15:RP110786. doi: 10.7554/eLife.110786 (PMC13268647; doi:10.7554/eLife.110786)
Supplement: Figure 4—source data 1. [file elife-110786-fig4-data1.docx]

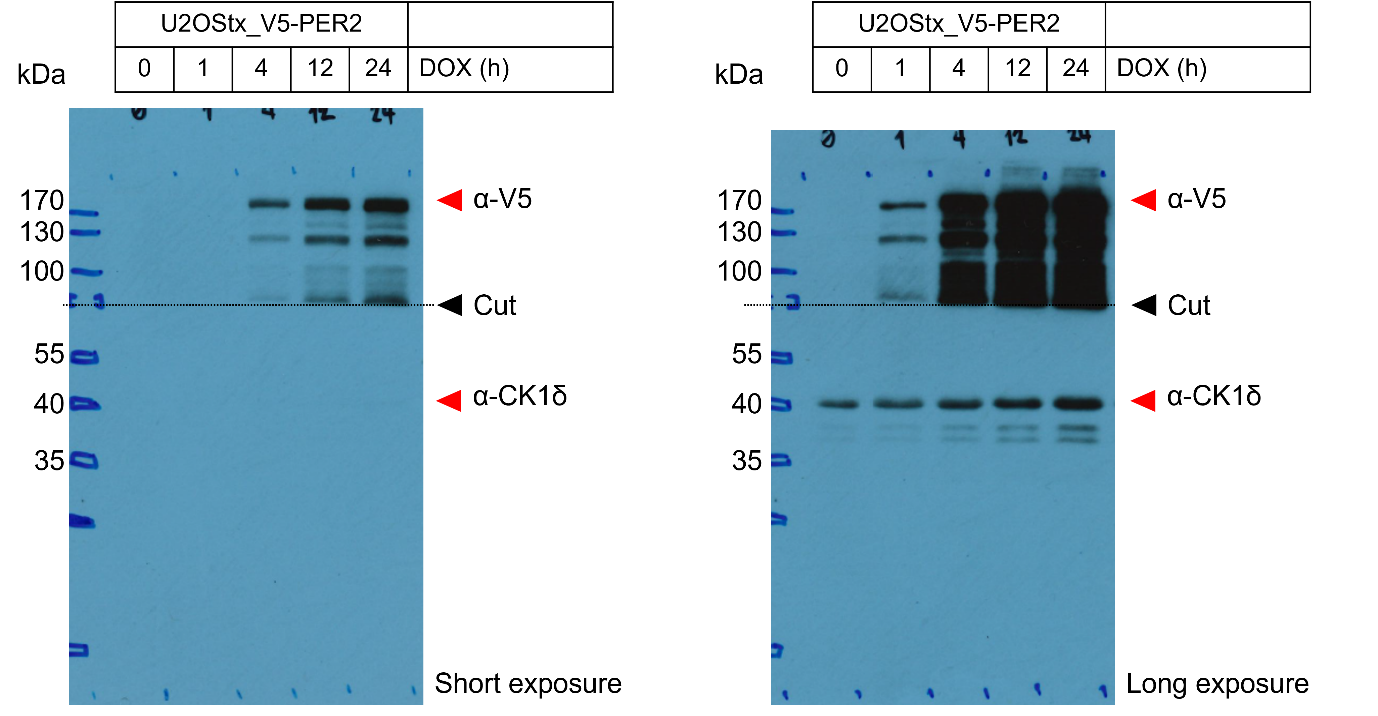


**Figure 4A – Source Data 1.** Original film corresponding to Figure 4A. U2OStx_V5-PER2 cells were induced with DOX and protein samples were taken 0, 1, 4, 12 and 24 h post-induction for immunoblotting. The top half of the blot was decorated with anti-V5 antibody to detect overexpressed V5-PER2 and the lower half was decorated with anti-CK1δ antibody to detect endogenous CK1δ. Both short and long exposures were taken from the same membrane.


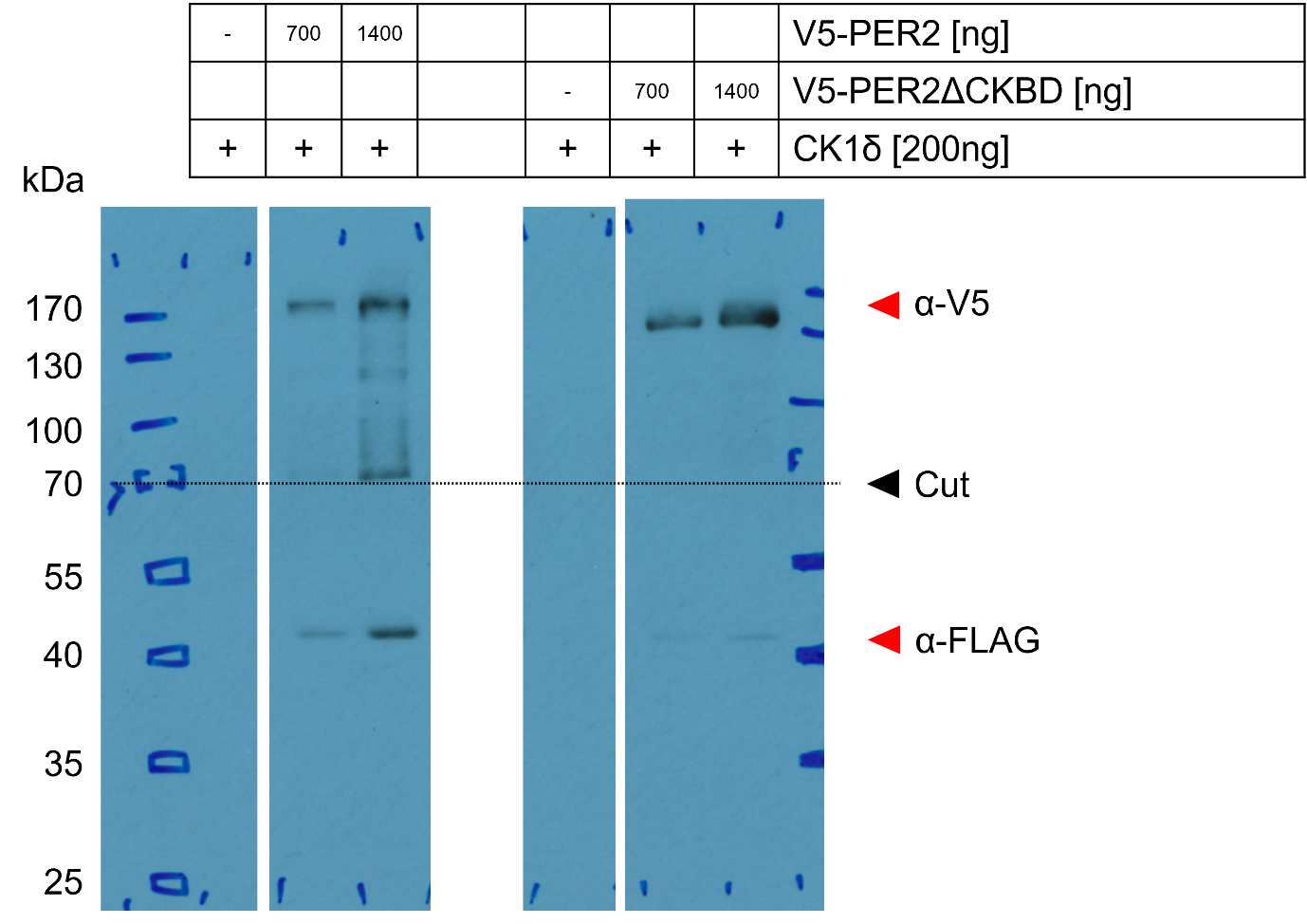


**Figure 4B – Source Data 1.** Original film corresponding to Figure 4B. HEK293T cells were transfected with plasmids as indicated, induced with DOX, and protein samples were for immunoblotting. The top half of the blot was decorated with anti-V5 antibody to detect PER2 and PER2ΔCKBD and the lower half was decorated with anti-FLAG to detect CK1δ.


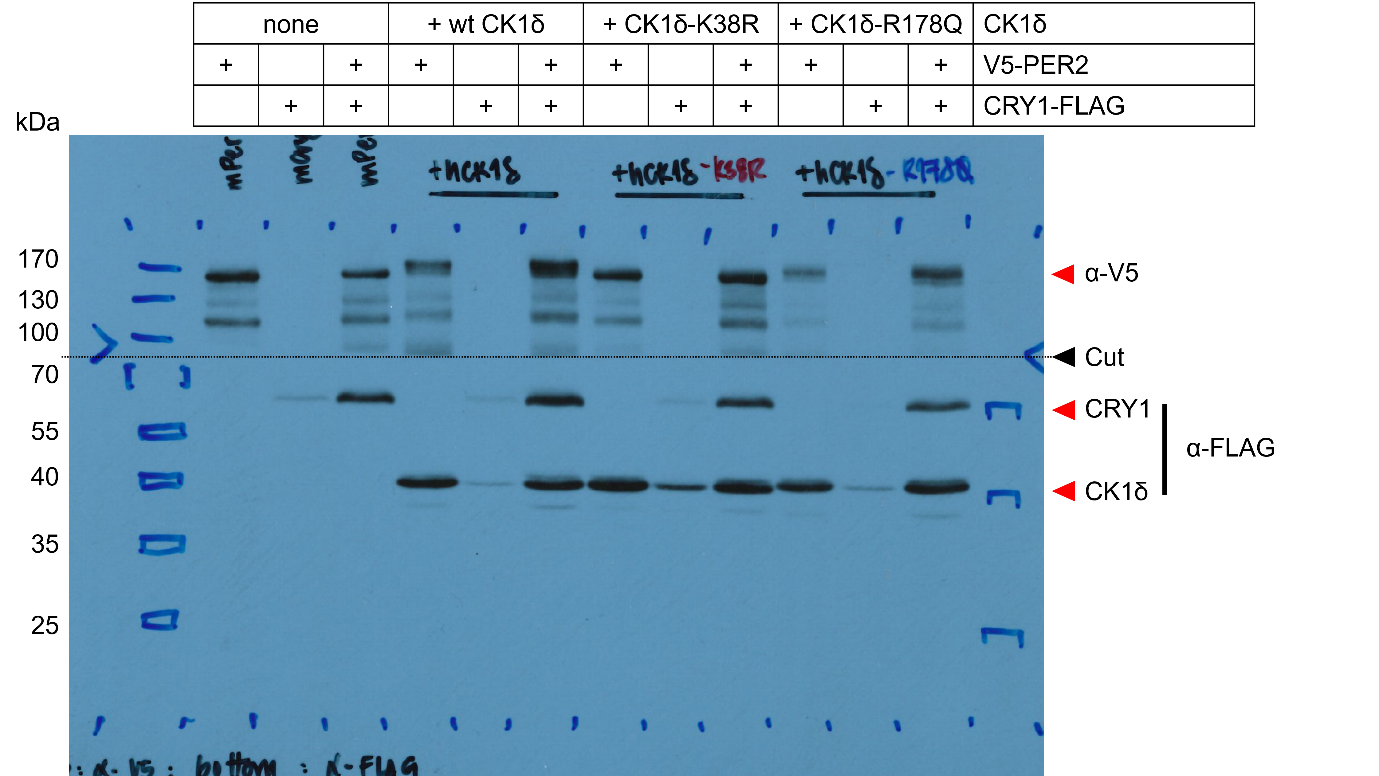


**Figure 4C – Source Data 1.** Original film corresponding to Figure 4C and Figure 4-figure supplement 1. HEK293T cells were transfected with plasmids as indicated, induced with DOX, and protein samples were extracted for immunoblotting. The top half of the blot was decorated with anti-V5 antibody to detect PER2 and the lower half was decorated with anti-FLAG to detect for both CRY1 and CK1δ. Left top and bottom boxes indicate cropped images used in Figure 4C.
