# Supplementary material for: Nuclear CK1δ as a critical determinant of PER:CRY complex dynamics and circadian period
Source: eLife. 2026 Jun 15;15:RP110786. doi: 10.7554/eLife.110786 (PMC13268647; doi:10.7554/eLife.110786)
Supplement: Figure 4—figure supplement 1—source data 1. [file elife-110786-fig4-figsupp1-data1.docx]

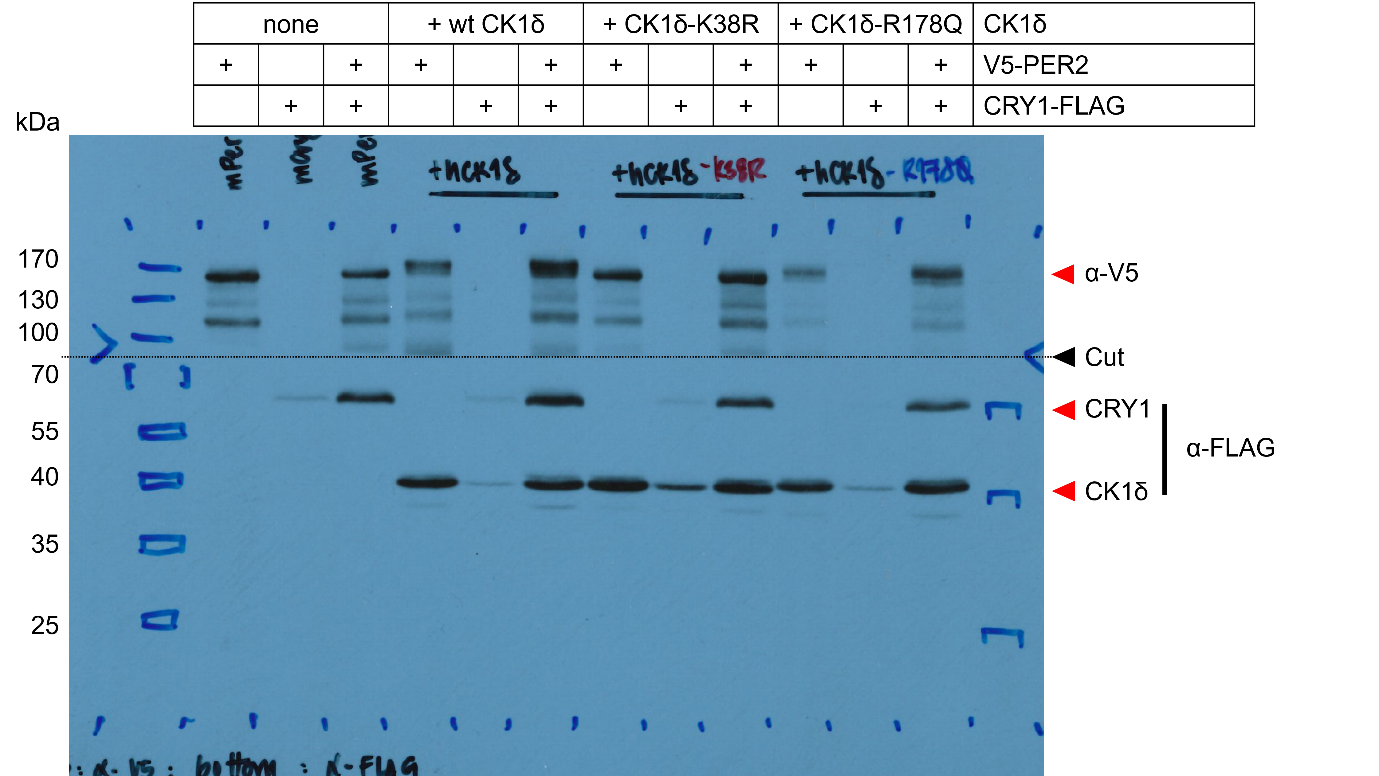


**Figure 4 – figure supplement 1 – Source Data 1.** Original film corresponding to Figure 4C and Figure 4-figure supplement 1. HEK293T cells were transfected with plasmids as indicated, induced with DOX, and protein samples were extracted for immunoblotting. The top half of the blot was decorated with anti-V5 antibody to detect PER2 and the lower half was decorated with anti-FLAG to detect for both CRY1 and CK1δ. Right top and bottom boxes indicate cropped images used in Figure 4 – figure supplement 1.
