# Supplementary material for: Nuclear CK1δ as a critical determinant of PER:CRY complex dynamics and circadian period
Source: eLife. 2026 Jun 15;15:RP110786. doi: 10.7554/eLife.110786 (PMC13268647; doi:10.7554/eLife.110786)
Supplement: Figure 6—source data 1. [file elife-110786-fig6-data1.docx]

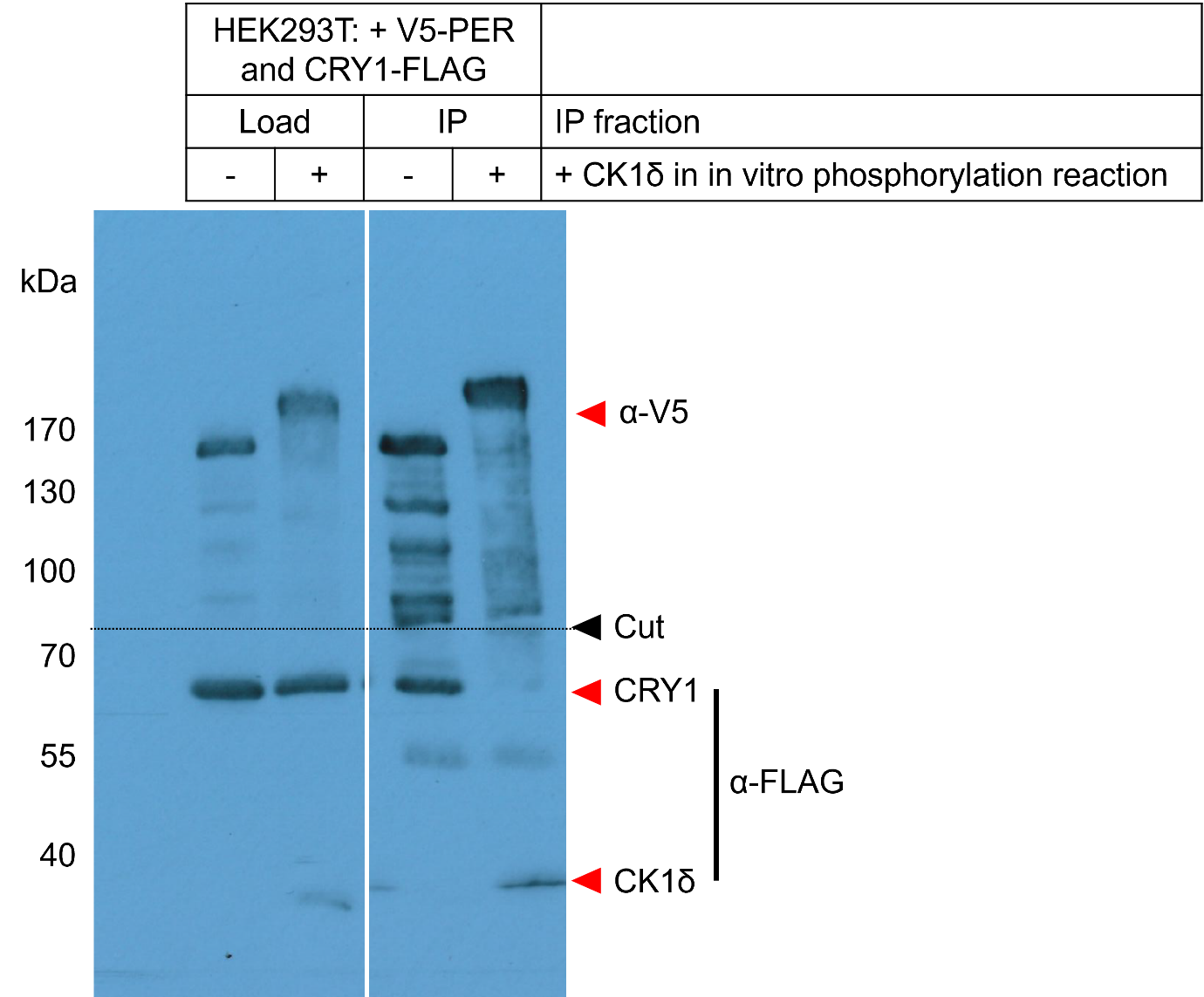


**Figure 6C, Source Data 1.** Original film corresponding to Figure 6C. HEK293T cells were transfected with PER2 and CRY1, protein was extracted and used for an *in vitro* phosphorylation reaction with CK1δ. The resulting extract was then used for a V5 IP to precipitate V5-PER2 and probe for interacting CRY1-FLAG. The top half of the blot was decorated with anti-V5 antibody and the lower half was decorated with anti-FLAG.


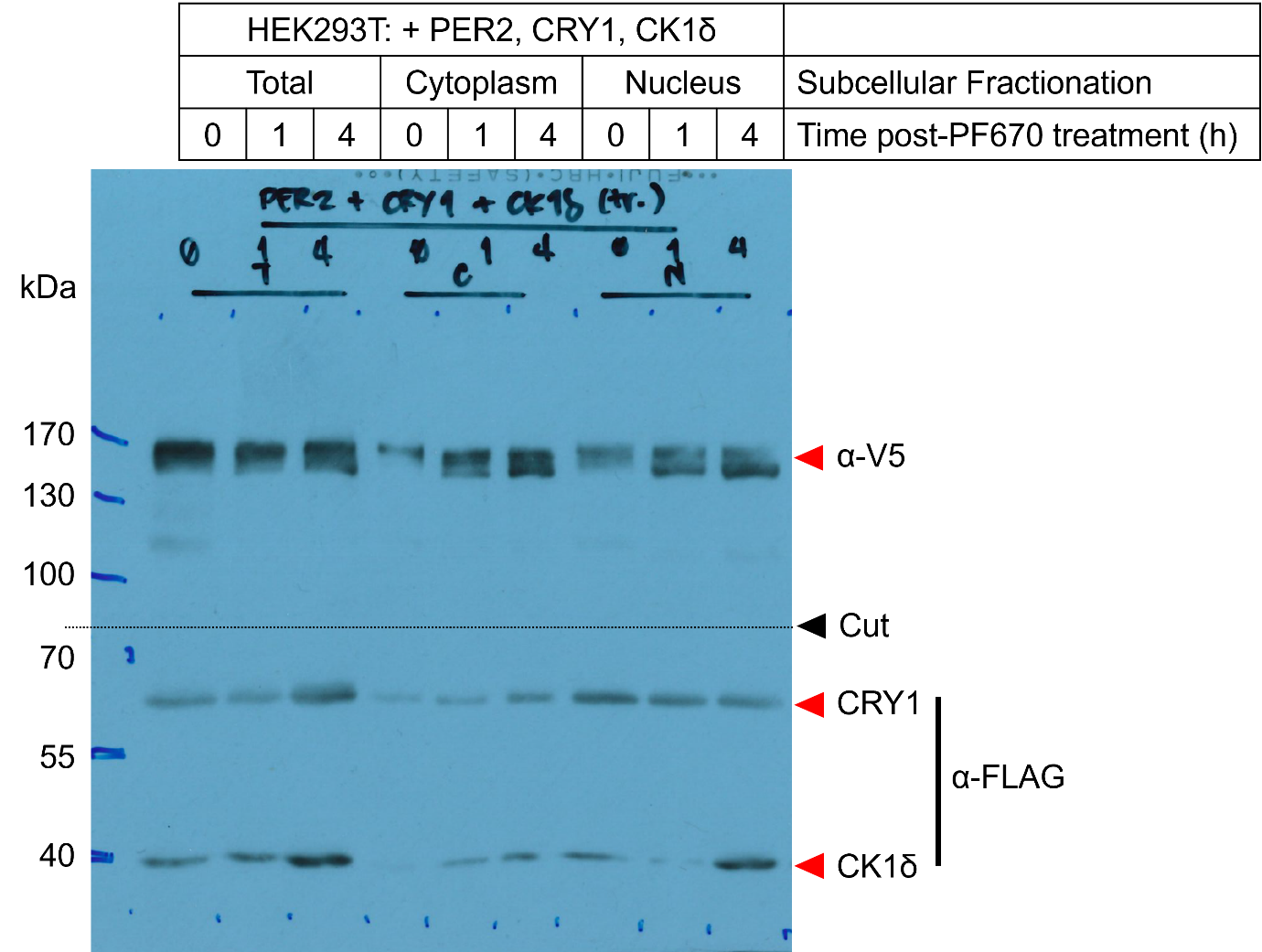


**Figure 6E, Source Data 1.** Original film corresponding to Figure 6E. HEK293T cells were transfected with PER2, CRY1, and CK1δ, treated with PF670, and underwent subcellular fractionation as indicated. The top half of the blot was decorated with anti-V5 antibody to detect PER2 and and the lower half was decorated with anti-FLAG to detect both CRY1 and CK1δ.


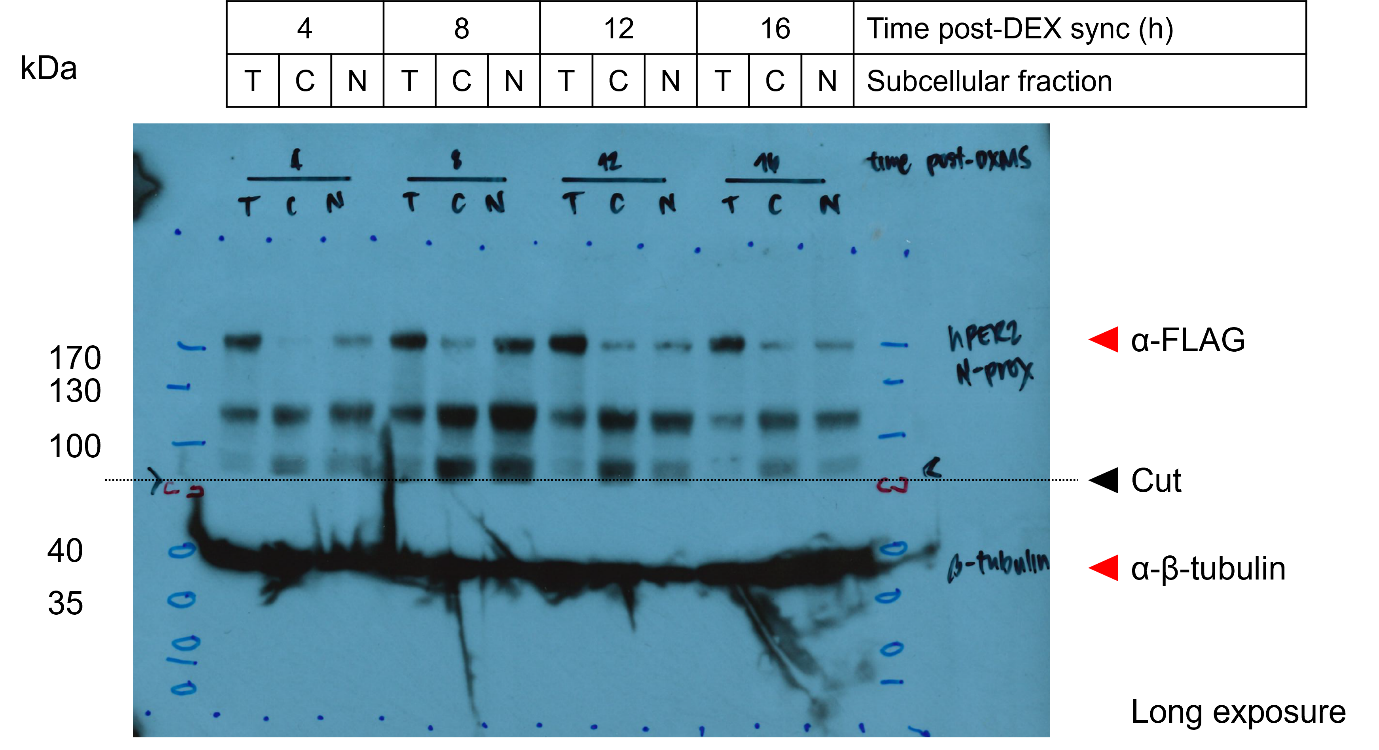


**Figure 6F, Source Data 1.** Original film corresponding to Figure 6F. U2OStx cells were synchronized with dexamethasone (DEX) and protein samples were taken 4, 8, 12, and 16 h post-synchronization for immunoblotting. The top half of the blot was decorated with anti-hPER2 antibody (in-house) and the lower half was decorated with anti-β-tubulin as a subcellular fractionation control.
