# Supplementary material for: Nuclear CK1δ as a critical determinant of PER:CRY complex dynamics and circadian period
Source: eLife. 2026 Jun 15;15:RP110786. doi: 10.7554/eLife.110786 (PMC13268647; doi:10.7554/eLife.110786)
Supplement: Figure 6—figure supplement 1—source data 1. [file elife-110786-fig6-figsupp1-data1.docx]

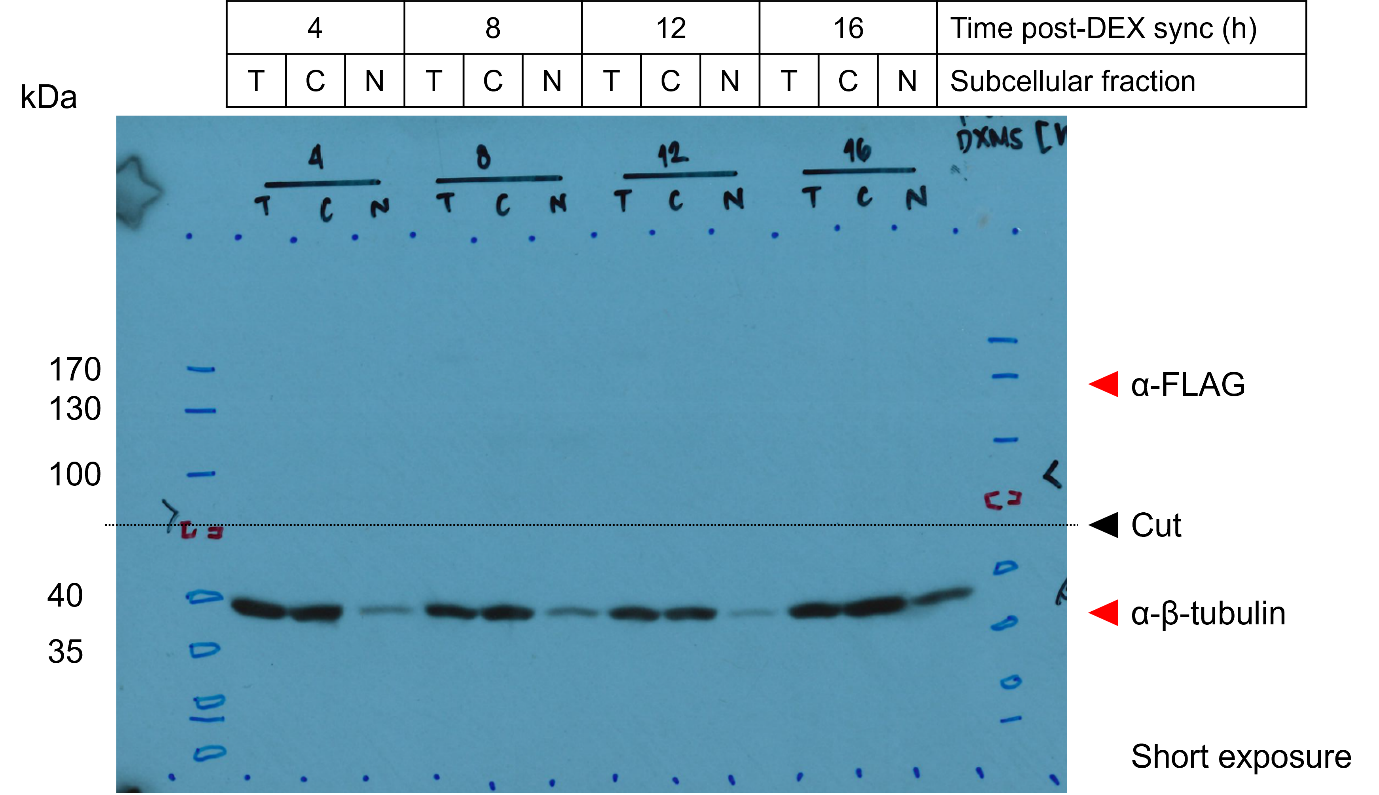


**Figure 6 – figure supplement 1D, Source Data 1.** Original film corresponding to Figure 6 – figure supplement 1D. U2OStx cells were synchronized with dexamethasone (DEX) and protein samples were taken 4, 8, 12, and 16 h post-synchronization for immunoblotting. The top half of the blot was decorated with anti-hPER2 antibody (in-house) and the lower half was decorated with anti-β-tubulin as a subcellular fractionation control.
