# Supplementary material for: Nuclear CK1δ as a critical determinant of PER:CRY complex dynamics and circadian period
Source: eLife. 2026 Jun 15;15:RP110786. doi: 10.7554/eLife.110786 (PMC13268647; doi:10.7554/eLife.110786)
Supplement: Figure 7—source data 1. [file elife-110786-fig7-data1.docx]

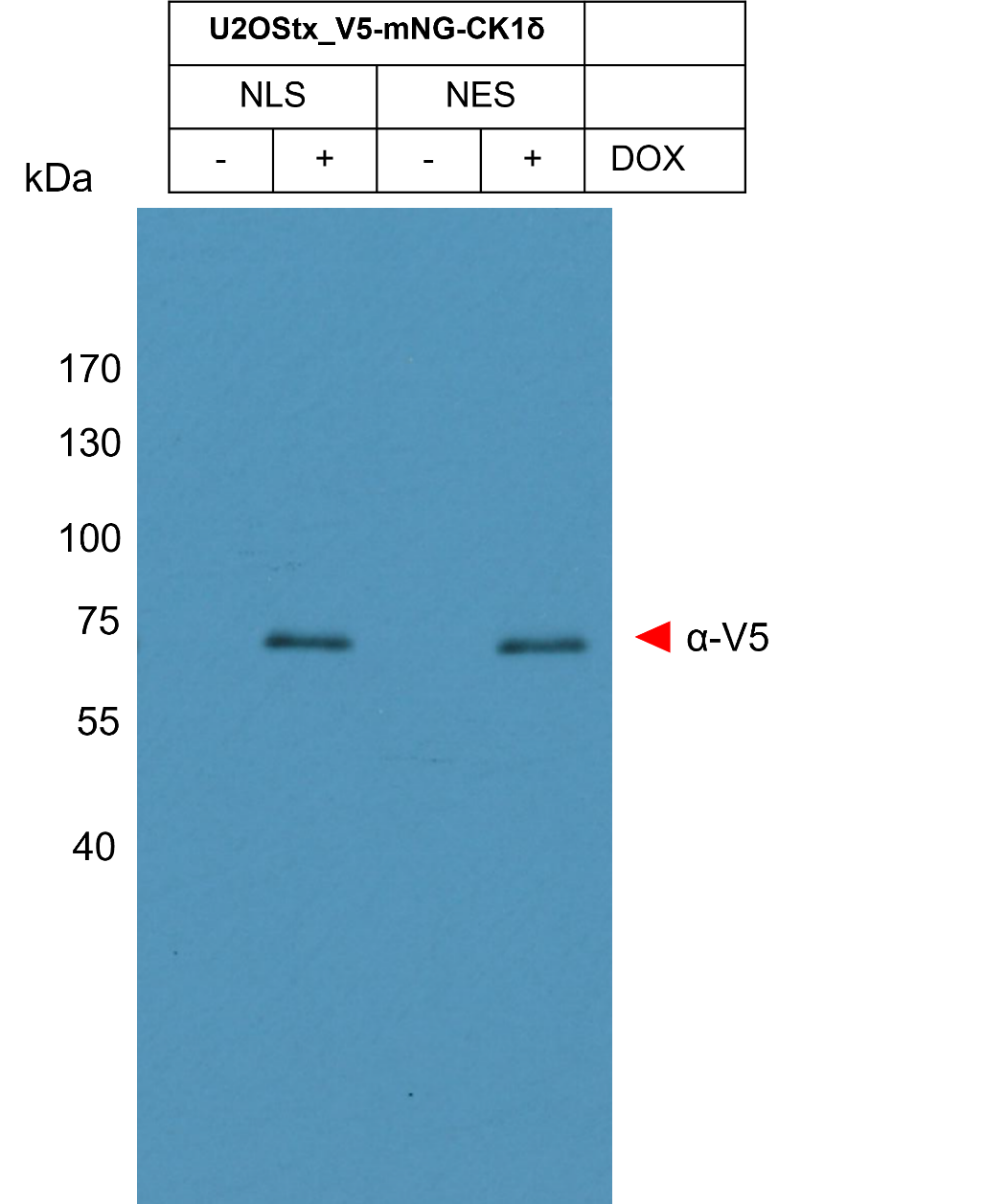


**Figure 7, Source Data 1.** Original film corresponding to Figure 7D. Stable U2OStx cell lines expressing inducible V5-NLS-mNG-CK1δ and V5-NES-mNG-CK1δ were generated, induced with DOX and protein was extracted for immunoblotting. The membrane was decorated with anti-V5 antibody.
