# Supplementary material for: Nuclear CK1δ as a critical determinant of PER:CRY complex dynamics and circadian period
Source: eLife. 2026 Jun 15;15:RP110786. doi: 10.7554/eLife.110786 (PMC13268647; doi:10.7554/eLife.110786)
Supplement: Peer review history

# Editor report: eLife Assessment

**DOI:** 10.7554/eLife.110786.2.sa0

**Authors:** Volker Dötsch

**Affiliations:** Goethe University Frankfurt Germany

**Keywords:** Solid, Valuable

This **valuable** study examines the subcellular dynamics of the mammalian circadian clock proteins PER2, CRY1, and CK1, providing **solid** evidence that CK1 modulates the PER2-CRY1 interaction and drives the cytoplasmic localization of PER2 complexes. This could play a key role in modulating transcriptional repression by PER2, CRY1, and CK that contributes to the molecular circadian clock. There are minor concerns regarding the overexpression of the clock proteins in this study.

[Editors' note: this paper was previously reviewed by another journal.]

# Referee report: Reviewer #2 (Public review)

**DOI:** 10.7554/eLife.110786.2.sa1

Summary:

This study aims to examine the effects of the subcellular localization of the mammalian clock protein PER2 and its dedicated binding partners CRY1 and the kinase CK1. Using a combination of transient transfection and a Dox-inducible expression system, they show that CRY1 promotes nuclear retention of PER2, and that phosphorylation of PER2 by CK1 promotes cytoplasmic localization and release of CRY1. Changes in complex assembly and subcellular localization could impact the transcriptional repressive function of the CK1-PER2-CRY1 complex in the molecular clock.

Strengths:

The study establishes a system of transient transfection and Dox-inducible expression that allows for strict temporal control of the presence of fluorescently-tagged clock proteins. This is essential to conduct time-lapse microscopy studies that determine changes in the apparent subcellular localization and stability of associated clock proteins. With the potential caveats of overexpression set aside, the authors make use of good controls and supplement cell-based work with in vitro experiments where possible. The discovery that phosphorylation of PER2 by CK1 in the nucleus leads to cytoplasmic localization of PER2 and PER2-CRY1 complexes is a new finding. Moreover, the apparent dissociation of CRY1 from PER2 after CK1 phosphorylation provides a potentially new mechanism by which the repressive activity of this complex could be regulated.

Weaknesses:

Overexpression of circadian clock components, normally expressed at low levels, could disrupt the stoichiometry of native interactions, Although the authors provide a reasonable rationale for the Dox-inducible approach and use appropriate controls throughout the experiments, there is still concern that overexpression of the components of this transcriptional repressive complex far exceed the concentration of the transcription factor they regulate, and this has not been taken into consideration here. In addition, the interesting discovery that CK1 phosphorylation of PER2 leads to dissociation of CRY1 has not identified the phosphorylation site(s) responsible for this, so the mechanism by which this occurs is still unknown. Still, this study provides some interesting hypotheses regarding CK1 regulation of PER2 and CRY1 that could drive future work in the field.

Comments on latest version:

This manuscript has already undergone two rounds of review at a reputable journal, and we have been provided with the previous reviewers' comments and the authors' responses. I am satisfied with the responses and changes to the manuscript made in these previous rounds of review and don't have any further experiments to suggest that wouldn't represent significant additional work.

# Author comment: Author response

**DOI:** 10.7554/eLife.110786.2.sa2

**Authors:** Fidel Emmanuel Serrano, Daniela Marzoll, Bianca Ruppert, Axel CR Diernfellner, Michael Brunner

**Affiliations:** Heidelberg University Heidelberg Germany; Heidelberg University Heidelberg Germany; Heidelberg University Heidelberg Germany; Biochemistry Center, Heidelberg University Heidelberg Germany; Heidelberg University Heidelberg Germany

[These author responses are to reviews from another journal.]

**Reviewer #1:**

This manuscript investigates the behaviour of a variety of clock proteins in cultured cells when epitope tagged and transiently expressed and try to draw general implications for endogenous function of circadian clock proteins.

Clock proteins are expressed at low levels in most cells, and so the clock interacting proteins (other kinases, phosphatases, ubiquitin-conjugated enzymes, etc.) are likewise probably at low abundance. Over-expression of one or two or even three components of a multicomponent system is going to produce odd and obscure non-physiological imbalances. The authors do not extend detailed study of these imbalances to more physiologic levels so the importance of their observations to clock function is not clear, and importantly, they are not tested in more biologically relevant models.

To study the function of components within a system, the steady state must be perturbed in one way or another. This can be achieved through pharmacological treatment, mutagenesis, downregulation, or overexpression. Such interventions are inherently non-physiological, and the relevance of the resulting observations must therefore be carefully validated.

In our study, the purpose of PER2 overexpression was to investigate its subcellular dynamics in the absence and presence of CRYs, specifically CRY1. This is far less trivial than it might appear at first glance, because our data clearly show that PER2 overexpression triggers, within 24 h, the accumulation of endogenous CRY1 (Fig. 1A), due to PER2-mediated stabilization of CRY1 (Fig. 4). PER2 overexpression also induces the accumulation of endogenous PER1, CK1, and BMAL1 (Fig. 2).

This effect was not considered in previous studies, such as Yagita et al. (2002), in which PER2 subcellular localization was assessed at a single time point following transient transfection. Yagita et al. found roughly equal proportions of cells with PER2 exclusively in the nucleus, exclusively in the cytoplasm, or distributed between both compartments. Such extreme cell-to-cell variability cannot be explained solely by PER2’s shuttling dynamics, as that would imply synchronous export in one cell and synchronous import in another.

Our time-resolved analysis of DOX-induced PER2 expression strongly suggests that the variability reported by Yagita et al. reflects a heterogeneous population of unsynchronized cells at different temporal stages along a trajectory from cytoplasmic PER2 (unbound) to nuclear PER2 fully saturated with CRYs (bound), owing to stabilization of endogenous CRYs. Similarly, Öllinger et al. (2014) analyzed PER2 nuclear export in cells constitutively expressing PER2-Dendra. Under such steady-state conditions, PER2-Dendra is already in complex with endogenous CRYs. The slow export rate and lack of dependence on additional CRY1 expression therefore likely reflect export of the complex, which is intrinsically slow.

Thus, prior to our work, no data on the true shuttling dynamics of PER2 were available.

Importantly, our results show not only that CRY1 promotes nuclear accumulation of PER2 (as reported by Öllinger et al.) but also that, conversely, PER2 promotes cytosolic accumulation of CRY1, depending on their expression ratio. Since CRY1 is predominantly nuclear and PER2 predominantly cytosolic, and because a PER2 dimer can bind one or two CRY1 molecules, our data suggest that the shuttling equilibrium depends on PER2 saturation state: a PER2 dimer bound to one CRY1 remains cytosolic, whereas a dimer bound to two CRY1 is nuclear.

These observations are novel and have not been reported previously. They were only possible through time-resolved analysis of overexpressed proteins.

A number of the findings are confirmatory rather than novel - the phosphorylation-regulated nuclear-cytoplasmic shuttling of CK1 and PER proteins is long known, and it's not clearly stated what is novel here.

We acknowledge prior work by Milne et al. (2001), who showed that kinase-dead CK1 is predominantly nuclear and that prolonged treatment with leptomycin B (16 h) enhances its nuclear localization. We cite this study at the beginning of the relevant paragraph. While we confirm these earlier observations, our work extends them in several important and novel ways:

(1) Rapid dynamics of CK1 localization – We show that pharmacological inhibition of CK1 with PF670 induces rapid (within 1 h) depletion of CK1δ from the centrosome, accompanied by nuclear accumulation and elevated CK1δ levels. These kinetics have not previously been reported. We also show that proteasome inhibition with MG132 enhance centrosomal staining, indicating that centrosomal binding sites are not saturated. Together, the data show that CK1δ equilibrates rapidly between its binding partners.

(2) Integration of localization with protein stability – We relate the known localization patterns of WT CK1 and the kinase-dead mutant K38R to CK1 degradation dynamics and further compare them to the tau-like kinase mutant CK1δ-R1178Q. This integration of subcellular localization data with turnover mechanisms provides new mechanistic insight.

(3) Comprehensive regulatory model – In the revised manuscript, we now include a schematic summarizing how CK1δ is posttranslationally regulated via subcellular shuttling, nuclear degradation, and dynamic interactions with binding partners (Figure EV5C). To our knowledge, such a comprehensive view of CK1δ regulation, linking localization, stability, and partner association, has not been presented before.

We believe these additions clearly distinguish our findings from prior reports and highlight the novel aspects of our study.

The formation of PER and CRY and CK1 complexes likewise is well established. The finding that formation of multiprotein complexes stabilize otherwise unstable over-expressed proteins is interesting but not novel.

We fully agree that the existence of PER–CRY–CK1 complexes is well established. It is also known that PER2 stabilizes CRY1 by occupying the FBXL3 binding site and that CRY1 promotes the nuclear accumulation of PER2. We do not present these established interactions as novel findings.

Our novel contribution, as outlined above, is the discovery that the shuttling and subcellular localization of PER2 and CRY1 are mutually dependent on their expression ratio. Specifically, we show for the first time that the steady-state shuttling distribution PER2 alone is cytosolic due to its rapid nuclear export wherease CRY1 is predominantly nuclear (known). Given that CRY1 facilitates the nuclear import of PER2 (known) and that a PER2 dimer can bind either one or two CRY1 molecules, our data showing that cytoplasmic PER2-CRY1 foci contain less CRY1 than nuclear foci lead us to conclude that cytoplasmic PER2 complexes contain one CRY1 molecule, while nuclear complexes contain two.

This model provides a mechanistic explanation for the distribution of PER2 between the cytosol and nucleus and for the relatively lower cytosolic CRY1 levels. Moost importantly, we further show (for the first time) that CK1-mediated phosphorylation of PER2 displaces CRY1. This phosphorylation event would produce PER2 dimers with one or no CRY1 bound, promoting their export to the cytosol. We believe this represents a novel and potentially important mechanism for regulating circadian clock function.

The results from many of the imaging assays are not quantitated, and the figures often show single cells. It's hard to draw statistical significance from these.

The phenotypes we report here are result of multiple technical and biological replicates (n >3). Image analysis and statistical analysis was performed when required. We show additional examples in the EVs.

There are a number of phenomena seen whose physiological relevance is unclear. In figure 1, forced over-expression of CRY1 and PER2 leads to formation of nuclear foci. It is unlikely these foci form at non-overexpressed levels, and so the general interest and relevance is not high nor investigated. This reduces the impact of the finding.

It has been shown that PERs and CRYs do not form thermodynamically stable, large (detectable) foci under physiological conditions, as we have stated in the manuscript. Whether these proteins have the propensity to form smaller, more dynamic structures of physiological relevance is an interesting question that could be explored elsewhere, but it is not relevant to our study. In our work, these foci are simply convenient markers for analyzing the interaction and subcellular (co)localization of clock proteins under investigation. In the revised version, we have kept the analysis of these foci and the discussion of their potential relevance to a minimum in order to avoid confusion and unnecessary discussions.

The finding that CK1δ is keep in the dephosphorylated state by binding to PER has been established previously by Johnson and colleagues and should perhaps be mentioned (Qin JBR 2015 doi: 10.1177/0748730415582127).

There is clearly a misunderstanding here. Qin et al.’s data show that, in a cell-free system, CK1ε phosphorylates PER2 and also autophosphorylates its C-terminal tail (autoradiograph, Fig. 1E).

However, because PER2 phosphorylation is carried out by CK1ε that is tightly anchored to PER2, there is competition between PER2 phosphorylation and tail autophosphorylation. As a result, the kinetics of tail phosphorylation are slower (Fig. 3B and quantification in C) than those observed with free CK1ε (as seen in the presence of the p53 substrate, Fig. 3A,C). We believe that his is also happening in the cell.

**Author response image 1.** 

Our data, in contrast, address a different point. It has been known from the Virshup lab for decades that CK1δ/ε undergo futile cycles of (auto)phosphorylation and dephosphorylation, resulting in an active, dephosphorylated kinase in cells because cellular phosphatases are more efficient than CK1 autophosphorylation. We now show that CK1δ is also efficiently dephosphorylated when bound to PER2 (Fig. 3). Nevertheless, despite dephosphorylation of PER2-bound CK1δ, PER2 itself becomes hyperphosphorylated, indicating that cellular phosphatases act differently on these two substrates. To clarify this point, we inhibited phosphatases with calyculin A (CalA). Under these conditions, both PER2 and PER2-bound CK1δ became efficiently hyperphosphorylated (new Fig. 3).

The degradation of kinase-active but not inactive CK1 is only shown here with 50-fold overexpressed protein so it's interesting, but the relevance to circadian biology is not made clear. The fact that over-expressed CK1 is degraded primarily in the nucleus is interesting, but needs further characterization - is this affected by the epitope tag? Is it true of endogenous CK1 or only over-expressed CK1? Is this not seen with e.g. other forms of CK1, e.g. lacking the C-terminus?

The observation that unassembled kinase is rapidly degraded is most clearly demonstrated by overexpression experiments. However, Fig. 3 shows that overexpression of CRY1 and PER2 leads to the accumulation of elevated levels of endogenous CK1δ (untagged), indicating that endogenous kinase is likewise degraded in the absence of a stabilizing binding partner. In addition, we present data showing that overexpression of tagged CK1δ reduces the levels of endogenous, untagged CK1δ, further supporting the conclusion that unassembled endogenous CK1δ is unstable and subject to degradation.

Further characterization of the CK1 degradation pathway is of considerable interest and could form the basis of a separate study, particularly to identify the components that mediate activity-dependent nuclear export and activity-dependent nuclear degradation. The Δ-tail kinase is expressed at very low levels, although interpretation is complicated by the possibility that this reflects pleiotropic effects.

The final figure, showing that nuclear CK1 is the form responsible for shortening rhythms, is interesting. Is this because massive increases in nuclear CK1 alter PER, or BMAL/CLOCK, or proteasome activity?

Our data show that cells expressing either nuclear or cytosolic CK1 are viable, proliferate normally, and maintain a functional circadian clock. Therefore, overexpression of the kinase does not produce pleiotropic effects.

To assume it's due to PER phosphorylation is in disagreement with the studies of Meng et al. Neuron 2008 DOI 10.1016/j.neuron.2008.01.019.

The data are not in disagreement with Meng et al.; in fact, they align quite well. Meng et al. showed that CK1ε-tau shortens the circadian period, which we had also previously reported for CK1δ-tau-like (Marzoll et al., 2022). We now demonstrate that CK1δtau-like is enriched in the nucleus, contributing to its period-shortening phenotype. Furthermore, we show that active CK1δ (but not CK1δ-K38R) promotes cytoplasmic accumulation of PER:CRY complexes, consistent with PER2 degradation in the cytosol as described by Meng et al.

Taken together, these findings suggest that PER proteins acquire their CK1 in the nucleus, and this interaction determines the circadian period length. Following a time delay—set by the kinetics of PER2 phosphorylation—PER2:CRY complexes are exported to the cytosol along with their bound CK1, where they are subsequently degraded.

**Reviewer #2:**

Interactions between the circadian clock proteins PER1/2 with CK1d/e and CRY1/2 influence each of their stability, subcellular localization, and activity, as countless studies over the last two decades have shown. However, many questions still remain, especially in light of newer models of the transcription-translation feedback loop (TTFL) in which the repression phase relies on two distinct mechanisms, a phosphorylation-dependent displacement of the transcription factor by CK1-PER-CRY complexes from DNA early in repression, and a CRY1dependent sequestration of the transcription factor activation domain later in repression. In particular, questions remain about mechanisms triggering nuclear entry/export and activity of these proteins in the cytoplasm and nucleus.

Here, the authors utilize a system of induced and/or transient overexpression of proteins with or without with fluorophores to track subcellular localization, stability, and interactions. As the authors point out throughout the manuscript, the overexpression of these clock proteins often causes them to behave differently from the endogenous proteins. It looks as though the authors have done their best to account for these changes, and they have certainly been rigorous in pointing them out, but there is concern that some of the conclusions may be influenced by this overexpression. For example, the relevance of work related to the overexpression-dependent foci is unclear.

Same answer as to Reviewer 1: It has been shown that PERs and CRYs do not form thermodynamically stable, large (detectable) foci under physiological conditions, as we have stated in the manuscript. Whether these proteins have the propensity to form smaller, more dynamic structures of physiological relevance is an interesting question that could be explored elsewhere, but it is not relevant to our study. In our work, these foci are simply convenient markers for analyzing the interaction and subcellular (co)localization of the clock proteins under investigation. In the revised version, we have kept the analysis of these foci and the discussion of their potential relevance to a minimum in order to avoid confusion.

The findings that the stability of the kinase depend on localization, its intrinsic activity, and interaction with PER2 are interesting and important. Use of the CKBD deletion to show that CK1 stabilization depends on its anchoring interaction with PER2 is a nice touch. The authors bring up an excellent point that most of the potential phosphorylation sites on PER1 and PER2 have not been functionally characterized aside from the phosphoswitch mechanism. Their observation that CK1 eventually induces cytoplasmic localization of the CK1-PER-CRY1 complex and the release of CRY1 is intriguing. In particular, the finding that pretreatment of PER2 with CK1 in vitro blocked its ability to interact with CRY1 is very interesting. However, the absence of mechanistic data to explore this in more detail limits the impact of this conclusion. Using the system they have established here to identify the site(s) on PER2 and/or CRY1 that lead to this would help to solidify this work and increase the impact of this work. Overall, there are some interesting findings here but the inclusion of some competing viewpoints and mechanistic data would strengthen the impact of the work.

Major

(1) The characterization of the tau-like CK1 mutant R178C as less active than the wild type enzyme is not entirely correct-it is less active on the FASP region as described, but it has increased activity on S478 in the phosphodegron that is independent of inhibition from the FASP region (Gallego et al. PNAS, 2007 and Philpott et al. eLife, 2020). It is still possible that some of the period shortening effects of the mutant could arise from enhanced nuclear accumulation, but the oversimplified description of the mutant as less active should be corrected.

In the revised version, we discuss that the enhanced nuclear localization of the Tau-like kinase may contribute, at least in part, to period shortening, similar to how forced nuclear overexpression of wild-type kinase also shortens the period. We emphasize, however, that CK1 Tau is compromised in its priming-dependent activity, whereas its priming-independent activity is context-specific and enhanced toward the β-TrCP site.

(2) One of main conclusions from the paper, that CK1 induces cytoplasmic localization of the CK1-PER2-CRY1 complex and subsequent release of CRY1 would be strengthened significantly by identifying the phosphorylation site(s) responsible for the cytoplasmic localization of the complex and the release of CRY1. The system they have developed here seems ideal to identify these sites.

We fully agree with the reviewer. We substituted the known phosphorylation sites in PER2 surrounding the CRY-binding domain, but this had no effect on the phosphorylationdependent release of CRY1. Therefore, a more systematic analysis will be required, including the possibility that phosphorylations in CRY1 itself may contribute. To this end, we are generating PER2 and CRY1 variants in which all Ser/Thr residues are replaced by Ala. Using these constructs alongside the wild-type versions, we will by PCR systematically create hybrids in which specific regions containing phosphorylation sites are exchanged.

Nevertheless, this will require considerable time and effort, and we believe this investigation exceeds the scope of the present manuscript and will address it in future work.

(3) The concept of delayed release of CRY1 presented here is an interesting one. It's unclear why the authors have also not incorporated prior findings (Ukai-Tadenuma et al. Cell, 2012, Koike et al. Science, 2012) that peak levels of CRY1 are expressed in a later phase than CRY2, PER1, and PER2. It seems like figure EV6 should reflect the observation that CRY2 is the predominant cryptochrome present during early repression (Koike et al. Science, 2012).

The reviewer is absolutely right: the expression phases of CRY1, CRY2, PER1, and PER2 are important. I have recently discussed these issues in detail in a News & Views article in The EMBO Journal, commenting on a paper by Smyllie et al. In this News & Views article, I discuss that the presently available data suggest that CRY1 is always present throughout the circadian cycle and keeps circadian transcription partially repressed even at peak phases of expression. In the revised version, I refer to these publications, including those mentioned by the reviewer. However, I would like to keep the model presented in the supplementary figure as simple as possible and specifically focused on the work presented in this manuscript, rather than presenting a comprehensive conceptual model of the circadian clock.

(4) The model presented in figure EV6 and described throughout the text shows that PER-CRY complexes interact with CK1 in the nucleus, and not in the cytoplasm prior to nuclear entry. Prior work on endogenous protein complexes has shown that CK1-PER-CRY complexes exist in the cytoplasm very early on in the repression phase (Aryal et al. Mol Cell, 2017-ref. 14 in the manuscript). Work by Sancar and colleagues (Cao et al. PNAS, 2020) also shows with endogenous proteins that CK1d has a circadian pattern of nuclear entry (or possibly retention) concomitant with PER2 that is dependent on the presence of PERs and CRYs. Together, these data seem to be inconsistent with your model.

We think the data are not inconsistent. The recent Smyllie et al. paper in *EMBO Journal* shows that PER2 is present in both the cytosol and the nucleus at all times when it is expressed, but cytosolic PER2 is not saturated with CRY, which is more nuclear. Our data demonstrate that PER2 shuttles between the cytosol and the nucleus depending on its occupancy with CRYs (see schematic Fig. 1). Occupancy, in turn, depends on expression levels and binding affinities, including those of CRY2 and PER1. Consequently, PER2 complexes could shuttle continuously throughout the circadian cycle—either because they are not saturated with CRYs due to the balance between expression levels, freely available CRY, and binding affinity, or later in the cycle because CRYs are displaced by phosphorylation. If PER2 acquires casein kinase in the nucleus early in the cycle, it will shuttle out to the cytosol together with the bound CK1. We believe this does occur, but early in the circadian cycle the saturation of PER2 with casein kinase is likely to be very low due to the limited availability of CK1 in the nucleus. I am aware that not everyone will share this interpretation point by point, but discussing it in greater length and detail exceeds the scope of the present manuscript.

**Reviewer #3:**

This manuscript by Serrano and co-workers is a tight body of work that provides much needed insights into the regulation of clock proteins by CK1D, and into the regulation of CK1D itself. While the whole paper relies on artificial overexpression of chimeric/tagged proteins that may have significant differences in the function, the stability and subcellular distribution of the endogenous proteins they are suppose to model, this limitation was been clearly stated by the authors, and nevertheless their study still provides important insights.

While the authors have specified which Ck1d isoform (Ck1d1) they are overexpressing in their model cell lines, they may have thought to consider that the overexpression of one Ck1 homologue may affect the endogenous expression of the other homologues and their isoforms, e.g. ck1d1 overexpression may cause an increase in Ck1d2 or Ck1e, which would in turn affect the conclusions.

We show in revised Fig. 3 that overexpression of CK1δ1 reduces the expression of endogenous CK1δ1/2. This is consistent with our prediction that overexpressed and endogenous CK1 (including CK1ε) compete for the same stabilizing binding partners, leading to rapid degradation of unassembled kinases.

Moreover, the antibody they used for endogenous Ck1d (which is ab85320, also mentioned as AF12G4 but that is the clone number, not the catalogue number) is discontinued and its specificity against Ck1d1, Ck1d2 or even the highly identical Ck1e, has not been clearly demonstrated. We know from Fig 3 that it can detect Ck1d1 but it would be great if the authors would provide additional evidence for the specificity of this antibody, for example by overexpressing Ck1d1/Ck1d2/Ck1e to see really which "endogenous" Ck1 we are seeing.

Are the three bands for example seen in Fig 4A corresponding to the different isoforms? This simple experiment would reinforce the conclusions.

We show in the revised figure that the antibody recognizes CK1δ1 and CK1δ2, but not CK1ε. In U2OS cells, the antibody detects a single band (Figure); we do not know whether this represents predominantly one splice isoform or both, which are not resolved. However, this distinction is not relevant for our interpretation, because overexpression of tagged CK1δ1 reduces the expression of whichever endogenous kinase is present.

There are no minor comments, as the figures, the figure legends and main text are all of good quality and ready for publication.

**Reviewers’ Responses to Point-by-Point Response to Peer Review**

**Referee #1:**

I appreciated the additional efforts by the authors to improve the manuscript. Unfortunately, the underlying approach of forced over-expression remains artifact-prone, and has been largely supplanted by readily available knockin and targeted mutagenesis methods. Over-expression may give clues, but I think more rigorous mechanistic validation is needed to make this compelling. I cannot support publication of this manuscript.

**Referee #2:**

In their response to reviewers, the authors make the valid point that the steady state of a system is usually perturbed to study it. In this study, they have used overexpression of the clock proteins PER2, CRY1 and CK1 to study their effects on subcellular dynamics and stability. In justifying this choice, they refer to several papers that similarly overexpressed at least one of these components, stating that their time-resolved approach brings novel insights. However, there is a missed opportunity here to translate any lessons learned from overexpression studies to a system where the proteins are expressed at physiological levels and stoichiometry.

The authors reply to reviewer 1 stating that they conclude PER proteins acquire CK1 in the nucleus, but this does not account for other studies showing an apparent PER-CK1 complex in the cytoplasm during the early phases of repression and/or a pattern of PER-dependent nuclear entry of CK1 (Lee et al. 2001, Cell; Aryal et al. 2017 Mol Cell; Cao et al. 2021 PNAS). Given that all 3 of these studies were done with native expression levels, it seems incumbent upon the authors to demonstrate that their conclusions from the overexpression study are physiologically relevant by translating them in some way to a more native system. This also addresses a point made by reviewer 2, major concern 4 that was not satisfactorily addressed by the authors. Perhaps they could validate their hypothesis of PER shuttling and interactions with CK1 or CRY1 that alter this in a native system similar to Aryal or Cao et al. with the use of nuclear export inhibitors?

The response to reviewer 2, major concern 1 is thoughtful and much appreciated. However, simplifying the effects of the tau mutation on CK1 as having a decreased rate on priming-dependent phosphorylation but not priming-independent is not quite true-the tau mutation also decreases the rate of priming-independent phosphorylation of S662 (in humans) (Philpott et al. 2020, eLife).

Other papers appearing in this journal seem to all include at least one major new mechanistic insight. Although the authors do a diligent job in characterizing the overexpressed proteins in this system, some of their conclusions are at odds with prior studies of the system in more native conditions, so the potential impact of this work is unclear. To verify these conclusions or test new ones (ie, that CK1 disrupts PER-CRY1 interactions), they should use their insights to generate mutations or make perturbations in a native system and demonstrate that they still hold.

**Referee #3:**

The authors have adequately addressed the reviewers' comments, and it is my opinion that the manuscript is ready for publication. It is true, as previously mentioned by other reviewers, that the evidence presented rely on overexpression, which for the other reviewers seem to preclude publication. However, I find this to be a too strict opinion.

If the authors had indeed provided evidence using crispr-cas9-mediated genetic manipulation and tagging/mutating endogenous genes for all their experiments, thereby providing more physiological evidence of how clock proteins interact, they would probably have submitted their manuscript to an alternative journal with a higher impact.

As it stands, it is my opinion that, considering the evidence and limitations of the study, this manuscript is a good match for the journal.

**Author Rebuttal:**

Apologies for the delayed reply regarding our manuscript. In the meantime, we have added several new experiments which address the comments of the reviewers and more. These are now included as Figures 1C, EV3, 4D, 6E, 6F, EV6D, and EV7.

Figure 1C reinforces our observations from Figure 1B showing that induction of stably-integrated PER2 also results in accumulation of endogenous CRY1 at a timescale that is compatible with the gradual localization of overexpressed PER2 into the nucleus.

Figure EV3 addresses several technical comments from Reviewers #3 and #1, respectively: Figure EV3A shows that our CK1δ antibody recognizes CK1δ1 and CK1δ2, but not CK1ε. Figures EV 3B and C clearly show how overexpression of our transgenic CK1δ results in decreased endogenous CK1δ which further demonstrates the rapid turnover of active kinase.

Figure 4D addresses the comment from Reviewer #2. We clearly show that CK1δ is not kept in a dephosphorylated state by binding to PER. In addition to our direct comment to this point, Figure 4D shows that CK1δ regardless if it is expressed alone or in complex with PER2 is phosphorylated to a similar extent when the cells are treated with the phosphatase inhibitor CalA. As indicated in our direct response, we are rather more interested in the observation that cellular phosphatases act differently on PER2 compared to CK1δ despite being in the same PER:CK1δ complex (as shown by the clear stabilization of overexpressed CK1δ by co-expression of PER2).

Figures 6E, 6F, and EV6D demonstrate that our observations from overexpression systems are also observed in a more physiological context, addressing comments from Reviewers #1 and #2. Figure 6E shows that dephosphorylation of PER2 leads to its relocalization from the cytosol to the nucleus, while Figure 6F analyzes the subcellular localization of PER2 in the context of a functional circadian clock in U2OS cells. The latter demonstrates that PER2 is predominantly nuclear early in the circadian cycle, but redistributes to the cytosol at later time points. We included these experiments in response to the reviewer’s request for a more physiological context. Since we are not a mouse lab, this cell-based system represents the most physiological model we can provide. Figure 6F show the dynamics of endogenous PER2 from DEX-synchronized cells. At early timepoints, PER2 is predominantly nuclear likely due to the incorporation of CRY1 forming the PER:CRY complex. At later timepoints PER2 is redistributed between the cytoplasm and nucleus due to PER2 phosphorylation. Importantly, these results are consistent with and recontextualize the results from Liu et al. (Xie et al., PNAS, 2023) showing the hypophosphorylated PER2 at early timepoints post-DEX is predominantly nuclear and hyperphosphoryated PER2, that appear later post-DEX is predominantly cytoplasmic.

Finally, Figure EV7 provides a model how the subcellular distribution of CK1δ affects its assembly into the PER:CRY complex emphasizing how nuclear kinase enacts its role in the circadian clock.

**Response to Reviewers:**

We were disappointed by the categorical rejection of overexpression experiments. Without a specific discussion of why they would be inappropriate or not sufficient in the context of the work presented here, the blanket assertion that overexpression inevitably produces artifacts functions more as a rhetorical device than as a substantiated scientific argument. The fact that the term ‘physiological’ generally carries a positive connotation, whereas ‘overexpression’ is often perceived negatively, does not in itself justify the categorical rejection of experiments.

While we appreciate that some reviewers may personally prefer alternative strategies, we believe that the suitability of any approach must be evaluated in light of the specific biological questions being addressed. I cannot see a single specific point in the reviewers’ responses indicating that any of our experiments yielded artificial results. It is true that targeted knock-in and mutagenesis methods are available, however, these approaches are simply not suited to the questions raised in this manuscript. We also fully agree that, whenever possible, insights from overexpression studies should be validated in systems with a functional clock where proteins are expressed at physiological levels, which we did using U2OS cells, and noting the compatibility of our results with those in the literature using endogenously-tagged constructs. We have cited several recent studies that have investigated the subcellular distribution and circadian dynamics of endogenous or endogenously-tagged clock proteins in mice (Cao et al, 2021; Smyllie et al, 2022, 2016, 2025) and U2OS cells (Öllinger et al, 2014; Gabriel et al, 2021; Xie et al, 2023). While we cannot substantially expand on these previous observations, we confirm them in the revised version by demonstrating the nuclear-to-cytoplasmic relocalization of PER2 in U2OS cells over the course of a circadian cycle. In addition, we show that this process is, in principle, reversible: when CK1 is inhibited with PF670, overexpressed hyperphosphorylated cytosolic PER2 becomes dephosphorylated and accumulates in the nucleus.

Overall, we consider our approach not only complementary but also essential, as it enables us to address two key questions that would otherwise be difficult or even impossible to resolve:

(1) Mutual impact of PER2 and CRY1 on subcellular dynamics and the role of PER2 phosphorylation

Evidence from mouse liver (Cao et al, 2021), mouse SCN (Smyllie et al, 2022, 2025), and U2OS cells (Xie et al, 2023) indicates that a substantial fraction of PER2 remains cytoplasmic throughout its expression cycle, even in the presence of CRY1, which promotes PER’s nuclear import. The mechanisms underlying this cytoplasmic retention remain unclear, and no circadian function has yet been attributed to the cytosolic PER2 pool. Our study addresses how PER2 abundance, phosphorylation state, and stoichiometry relative to CRY1 govern their interaction and subcellular dynamics. This is physiologically relevant because PER1/2 and CRY1/2 proteins oscillate in expression and degradation out of phase, such that their concentrations, stoichiometry, and phosphorylation state vary systematically over the circadian cycle. Transient transfection and inducible overexpression combined with time-lapse microscopy are essential here, as they uniquely allow modulation of protein ratios and CK1δ levels and to resolve their dynamics.

Previous work established that CRY1 is nuclear and promotes PER2 nuclear accumulation (Smyllie et al, 2022). Our data extend this by showing that subcellular distribution is determined by the CRY1:PER2 ratio. While CRY1 alone is nuclear we show that PER2 alone is cytoplasmic due to rapid nuclear export. Mixed conditions reveal ratio-dependent shifts: at low CRY1-to-PER2 ratios, CRY1 relocalizes to the cytoplasm, whereas at high ratios, PER2 is retained in the nucleus. We explain this behavior by PER2 dimerization: dimers bound to two CRY1 molecules remain nuclear, while dimers bound to a single CRY1 localize to the cytosol. Such species can be expected to form in a physiological context depending on binding affinities and rhythmic expression levels and ratios across circadian time. Importantly, we show that CK1δ-mediated phosphorylation destabilizes PER2 and CRY1 interactions. From this, we infer that PER2 dimers with only a single bound CRY1 transiently form and accumulate in the cytosol, consistent with the lower CRY1-to-PER2 ratio we observe in the cytosol and that has also been reported in the SCN (Smyllie et al, 2025). With continued phosphorylation, PER2 dimers lose CRY1 altogether, while the released CRY1 accumulates in the nucleus. We suggest that this mechanism supports and extends the late repressive phase of the circadian cycle. Recent data show that hypophosphorylated PER2 is predominantly nuclear, whereas hyperphosphorylated PER2 is largely cytoplasmic in mouse liver (Cao et al, 2021; Xie et al, 2023), linking our data to a physiological context.

Taken together, these findings suggest a mechanism whereby stoichiometry, subunit composition, and CK1δ phosphorylation determine PER:CRY complex composition and localization. Crucially, these complexes and their dynamic relocalization could only be observed using inducible overexpression; knock-in strategies at endogenous levels would not be able to capture such states.

(2) Posttranslational regulation and subcellular homeostasis of CK1δ and impact on the clock

Previous work has shown that nuclear export of CK1δ depends on its kinase activity (Milne et al, 2001). Here, we further demonstrate that unassembled CK1δ is subject to degradation, with nuclear turnover accelerated by its catalytic activity. Thus, when evaluating the impact of CK1δ mutants on the circadian clock, one must consider not only kinase activity but also protein stability and subcellular distribution. We find that CK1δ availability for PER2 differs between cytosol and nucleus. In particular, nuclear CK1δ is limiting, and its abundance directly determines circadian period length. This is significant because subcellular CK1δ availability and posttranslational regulation have not previously been examined or incorporated into circadian clock models, as the kinase has been assumed to be non-limiting given its constant expression throughout the circadian cycle. Complex formation between CK1δ and PER is a well-established determinant of circadian timing, with CK1δ overexpression known to shorten period length. Our data explain why: the binding equilibrium between CK1δ and PER must be finely tuned. Previous studies suggested that PER associates with CK1δ in the cytosol and enters the nucleus as a PER:CRY:CK1δ complex (Lee et al, 2001; Aryal et al, 2017). Our data suggest that nuclear PER is not saturated with CK1δ. This is because levels of free, active CK1δ in the nucleus are low, owing to its rapid export or degradation by the nuclear proteasome, which limits its availability for PER binding.

Our overexpression studies support this mechanism. NES-tagged CK1δ overexpression does not alter circadian period length, because it fails to increase nuclear CK1δ levels: Each PER molecule can coimport only one kinase, a process already occurring in wild-type cells, and the few co-imported molecules rapidly equilibrate with the nuclear pool, where they are subject to export or degradation. In contrast, NLS-tagged CK1δ overexpression directly increases nuclear kinase abundance by antagonizing export, thereby enhancing PER binding and shortening circadian period. This multilayered regulation of CK1δ stability and localization and its consequences for PER2 availability would not have been revealed without targeted overexpression. Our findings therefore fill a key knowledge gap and remain fully consistent with previous studies (Lee et al, 2001; Aryal et al, 2017; Cao et al, 2021).

Conclusion: In sum, our findings are novel and physiologically relevant, aligning with data from mouse liver and SCN. While studies at strictly endogenous protein levels are important and necessary, perturbation of steady state is a standard strategy to uncover and observe novel mechanisms. Endogenous-level experiments would demand technically unrealistic systems (for example, even the simplest case, analyzing the subcellular dynamics of PER2 alone, would require cells lacking PER1, CRY1/2, and CK1δ/ε). Moreover, adjustment of PER2-to-CRY1 ratios cannot be achieved with stably integrated genes and of course not at physiological expression levels. Thus, inducible overexpression is not merely practical but currently the most feasible approach to dissect these dynamics. We complement our findings with data from U2OS cells with a functional clock, showing that the availability of nuclear CK1δ directly determines circadian period length. Although specific aspects of our extended model require further experimental validation, no published evidence contradicts it to date. Mechanistic discussions of the circadian clock have so far focused primarily on PER protein degradation. Our model broadens this perspective by incorporating CK1δ homeostasis, PER:CRY complex composition, subcellular localization, and their regulation by phosphorylation. In doing so, it provides a detailed framework to be critically tested and refined in future studies.
